# Thin is required for cell death in the *Drosophila* abdominal muscles by targeting DIAP1

**DOI:** 10.1038/s41419-018-0756-x

**Published:** 2018-07-03

**Authors:** Kumar Vishal, Simranjot Bawa, David Brooks, Kenneth Bauman, Erika R. Geisbrecht

**Affiliations:** 1 Department of Biochemistry and Molecular Biophysics, Kansas State University, Manhattan, KS 66506 USA; 2 Department of Cell Biology and Biophysics, School of Biological Sciences, University of Missouri-Kansas, Kansas City, MO 64110 USA

## Abstract

In holometabolous insects, developmentally controlled programmed cell death (PCD) is a conserved process that destroys a subset of larval tissues for the eventual creation of new adult structures. This process of histolysis is relatively well studied in salivary gland and midgut tissues, while knowledge concerning larval muscle destruction is limited. Here, we have examined the histolysis of a group of *Drosophila* larval abdominal muscles called the dorsal external oblique muscles (DEOMs). Previous studies have defined apoptosis as the primary mediator of DEOM breakdown, whose timing is controlled by ecdysone signaling. However, very little is known about other factors that contribute to DEOM destruction. In this paper, we examine the role of *thin* (*tn*), which encodes for the *Drosophila* homolog of mammalian TRIM32, in the regulation of DEOM histolysis. We find that loss of Tn blocks DEOM degradation independent of ecdysone signaling. Instead, *tn* genetically functions in a pathway with the *death-associated inhibitor of apoptosis* (*DIAP1*), *Dronc*, and *death-associated APAF1-related killer* (*Dark)* to regulate apoptosis. Importantly, blocking Tn results in the absence of active Caspase-3 immunostaining, upregulation of DIAP1 protein levels, and inhibition of Dronc activation. *DIAP1* and *Dronc* mRNA levels are not altered in *tn* mutants, showing that Tn acts post-transcriptionally on DIAP1 to regulate apoptosis. Herein, we also find that the RING domain of Tn is required for DEOM histolysis as loss of this domain results in higher DIAP1 levels. Together, our results suggest that the direct control of DIAP1 levels, likely through the E3 ubiquitin ligase activity of Tn, provides a mechanism to regulate caspase activity and to facilitate muscle cell death.

## Introduction

Programmed cell death (PCD) governs the development and homeostasis of multicellular organisms by controlling the patterning of adult body structures and the removal of obsolete or damaged tissues^1–4^. Mechanisms by which cells die can be divided into three types based upon morphological criteria^5^. Type I PCD, or apoptosis, is characterized by the upregulation of caspases accompanied by DNA fragmentation, membrane blebbing, and cell rounding^6^. Autophagy is referred to as Type II PCD and is distinguished by the presence of double-membraned autophagosomes^7^. Necrosis, the third type of PCD, is an inflammatory response characterized by cell swelling and rupture of the cell membrane^8^.

PCD is particularly evident during the metamorphosis of holometabolous insects, including *Drosophila*^3, 9^. The first signs of apoptosis are observed in embryogenesis and persist into the pupal stages where many larval tissues are remodeled in response to pulses of the steroid hormone 20-hydroxy-ecdysone (ecdysone)^9–11^. A pulse of ecdysone in late third instar larvae (L3) promotes the larval to pupal transition, whereas a second pulse triggers PCD around 12 h after puparium formation (APF)^12^. After old tissues are histolysed, newly formed tissues grow in the remaining 3.5 days before adult eclosion.

Most knowledge about tissue histolysis stems from the analysis of salivary gland or midgut tissue in pupal metamorphosis^9^. Salivary gland histolysis is regulated by the late ecdysone pulse^13–15^, whereby ecdysone binding to its receptors promotes the expression of early genes, including the transcription factors *Broad complex* (*Br-C*)*, E74 A*, and *E93*^16–19^. Subsequent activation of cell death genes, including *hid*, *reaper*, *Dronc, Drice*, and the *autophagy-related gene 1* (*Atg1*), result in the elimination of this tissue^9, 14, 15, 17, 20, 21^. While salivary gland histolysis is mediated by both apoptosis and autophagy^22^, midgut histolysis is triggered by the early ecdysone pulse and is primarily regulated by autophagy^23^. Less is understood about muscle remodeling during pupation, including the signaling pathways that control whether muscle cells are fated to live or die.

**Fig. 1:**
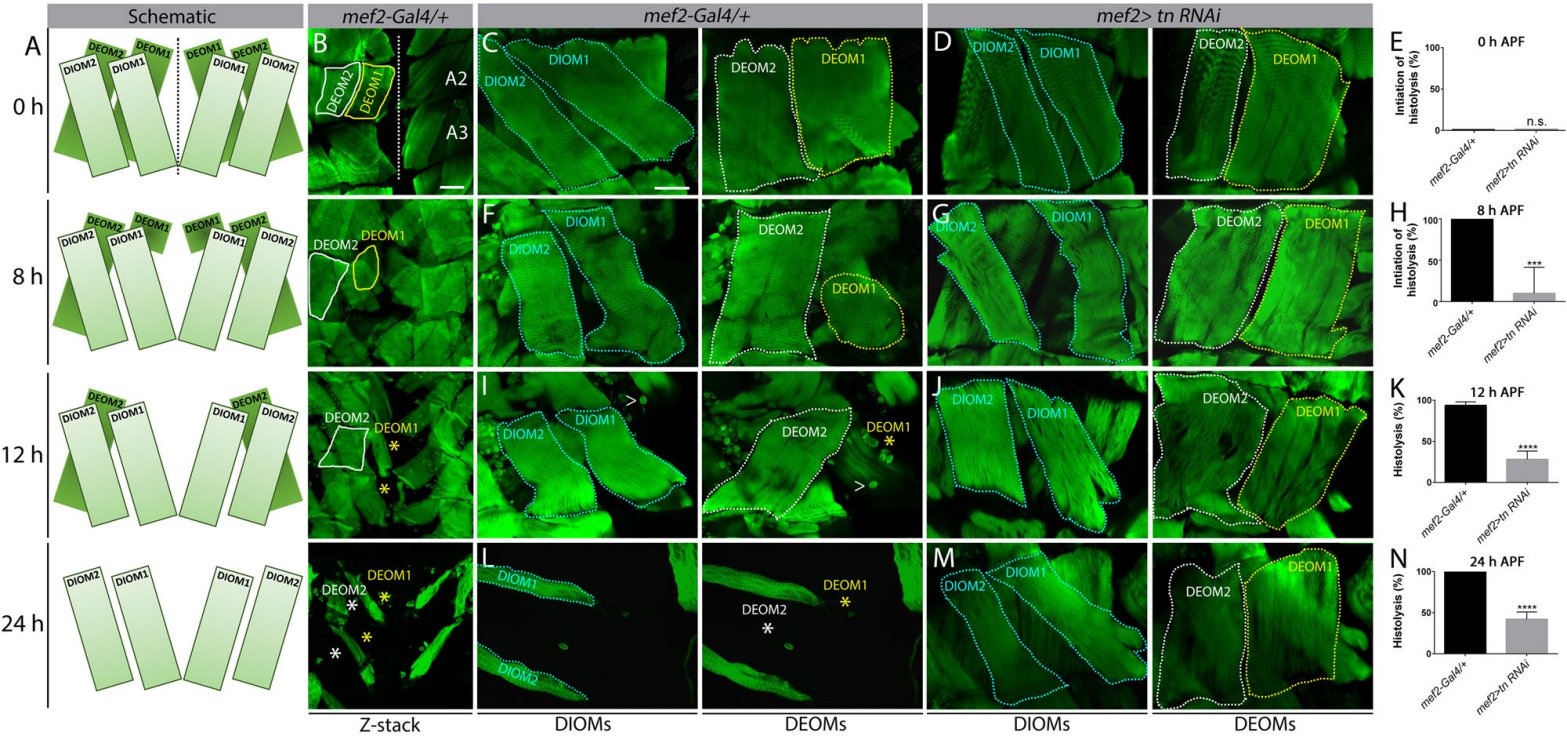
Tn is required for DEOM histolysis. **a** Schematic diagrams of the DEOMs during pupal development at 0, 8, 12, and 24 h APF. Dotted line denotes the midline. **b** Merged Z-stack images of DEOM histolysis that correspond to the same time points (**a**) in *mef2-Gal4/+* control muscles stained to visualize F-actin (green). DEOM1 (yellow solid line) and DEOM2 (white solid line) are both present at 0 h APF. In *WT* muscles, DEOM1 starts to disintegrate at 8 h APF and is gone by 12 h APF (yellow asterisk). DEOM2 disappears by 24 h APF (white asterisk). A2 and A3 denotes abdominal segments 2 and 3, respectively. **c**–**n** Representative images and quantification of DEOM muscle histolysis at 0, 8, 12, and 24 h APF in *mef2-Gal4/+* control or *mef2>tn RNAi* muscles stained with phalloidin (green). Substacks of single confocal planes separate out the DIOMs (cyan dotted lines) from the DEOMs. **c**, **f**, **i**, **l** DEOM1 (yellow dotted lines) and DEOM2 (white dotted lines) muscles degenerate (asterisks) by 24 h in control muscles. **d**, **g**, **j**, **m** However, reduction of *tn* by RNAi mostly blocks DEOM histolysis. **e**, **h**, **k**, **n** DEOM1 histolysis is not initiated at 0 h in *mef2-Gal4/+* control or *mef2>tn RNAi* muscles (**e**). By 8 h, all control DEOM1s have started to breakdown, while most of these muscles are still present in *tn RNAi* pupae (**h**). By 12 h (**k**) or 24 h (**n**), most DEOM1s are still intact in *tn RNAi* muscles. White carets indicate remnants of fat body tissue that stain positive for F-actin. Mean ± SEM (n.s., not significant, ****p < 0.001, ***p < 0.005). Scale bars, 100 µm (**b**); 50 µm (**c**, **d**, **f**, **g**, **i**, **j**, **l**, **m**)

*Drosophila* makes two sets of muscles during its life cycle, one in embryogenesis for larval movement and the other during pupation for adult life. During metamorphosis, most of the larval muscles are histolyzed and this pupal remodeling assures muscles are functional for adult-specific functions like flight and mating^24, 25^. Two sets of muscles that undergo remodeling during the pupal transition are the dorsal internal oblique muscles (DIOMs) and the dorsal external oblique muscles (DEOMs)^26, 27^. Both of these muscle groups are present in abdominal segments A1 to A5. The muscles closest to the midline are designated as DIOM1 or DEOM1, whereas more lateral muscles are classified as DIOM2 or DEOM2 (Fig. 1a, b). DIOMs fail to undergo histolysis and persist until adult stages, whereas the DEOMs are removed by PCD^26, 28^. DEOM1 histolysis is initiated by 8 h APF and the muscles are lost by 12 h APF. DEOM2 histolysis is delayed and is typically completed by 24 h APF^28–30^.

Destruction of the DEOMs is mediated solely by apoptosis^28^. Transmission electron microscopy studies show myonuclei containing condensed chromatin in dying DEOMs and ectopic expression of the anti-apoptotic protein p35 in DEOMs is sufficient to prevent muscle breakdown. In contrast, muscle-specific reduction of the autophagy gene *Atg1* has no effect^28^. Like salivary gland and midgut histolysis, ecdysone signaling mediates DEOM breakdown, which is evidenced by a reduction in caspase staining and the absence of muscle histolysis upon loss of the ecdysone receptor^28^.

The core machinery required for apoptosis is conserved among flies, worms, and mammals^2, 31^. The caspase family of proteins are the principal mediators of cell death and are present in most cells in an inactive form^32, 33^. Under normal conditions, caspase activity is blocked by the inhibitor of apoptosis (IAP) family of proteins to prevent cell death^34, 35^. In *Drosophila*, Dronc (Caspase-9) acts as the principal initiator caspase and is inhibited by DIAP1^36–38^. Upon receiving a cell death stimulus, the IAP antagonists Reaper, Hid, and Grim (RGH) promote the degradation of DIAP1, resulting in Dronc release^39–43^. A Dronc–Dark complex form the apoptosome to activate the executioner caspases Drice and Dcp-1, which cleave cellular substrates to promote cell death^44, 45^.

Herein, we have discovered that Tn acts in a pathway with DIAP1 and Dronc to regulate abdominal muscle breakdown. *tn*, also called another B-box affiliate (*abba*), is homologous to mammalian TRIM32 and is characterized by an N-terminal RING domain followed by six NHL repeats at the C terminus^46, 47^. The RING domain provides E3 ubiquitin ligase activity, whereas the NHL repeats are predicted to facilitate protein–protein interactions. Pleiotropic roles exist for the ubiquitously expressed TRIM32 protein in regulating muscle physiology, muscle regeneration, and tumor suppression^46, 47^. Our results here demonstrate a role for Tn in controlling the fate of muscle cells, acting as a switch to control whether muscle cells live or die.

## Results

### Tn is required for muscle histolysis and is not an ecdysone target

We previously showed that Tn is required for myofibril stability and costamere integrity in *Drosophila* larval muscles^48^. However, the late pupal lethality of *tn* mutants suggested that Tn may be required during pupal metamorphosis. Indeed, targeting *tn RNAi* in the musculature using the Gal4/UAS system produced defects in abdominal muscle histolysis.

At 0 h APF, all DEOMs were present in both control (*mef2-Gal4/+*) and experimental (*mef2*>*tn RNAi)* genotypes (Fig. 1c–e). Around 8 h APF, smaller, rounded muscles indicated the onset of DEOM1 histolysis in controls (Fig. 1f, h), while DEOM1 was still fully intact upon a decrease in Tn (Fig. 1g, h). By 12 h APF, DEOM1 was absent in nearly all *mef2-Gal4/+* individuals (Fig. 1i, k), while loss of DEOM1 was observed only in approximately 28% of *tn RNAi* abdominal segments (Fig. 1j, k). Complete histolysis of DEOM1 (and DEOM2) were apparent in controls at 24 h APF (Fig. 1l, n). In contrast, a partial block in degradation was observed in DEOMs with disrupted Tn function at 24 h APF (Fig. 1m, n). This block in DEOM histolysis was verified in *tn*^*ΔA*^*−/−* mutants (*tn−/−*) and an additional *tn RNAi* line (Fig. S1). Thus, we conclude that the abrogation of Tn results in impaired muscle histolysis.

**Fig. 2:**
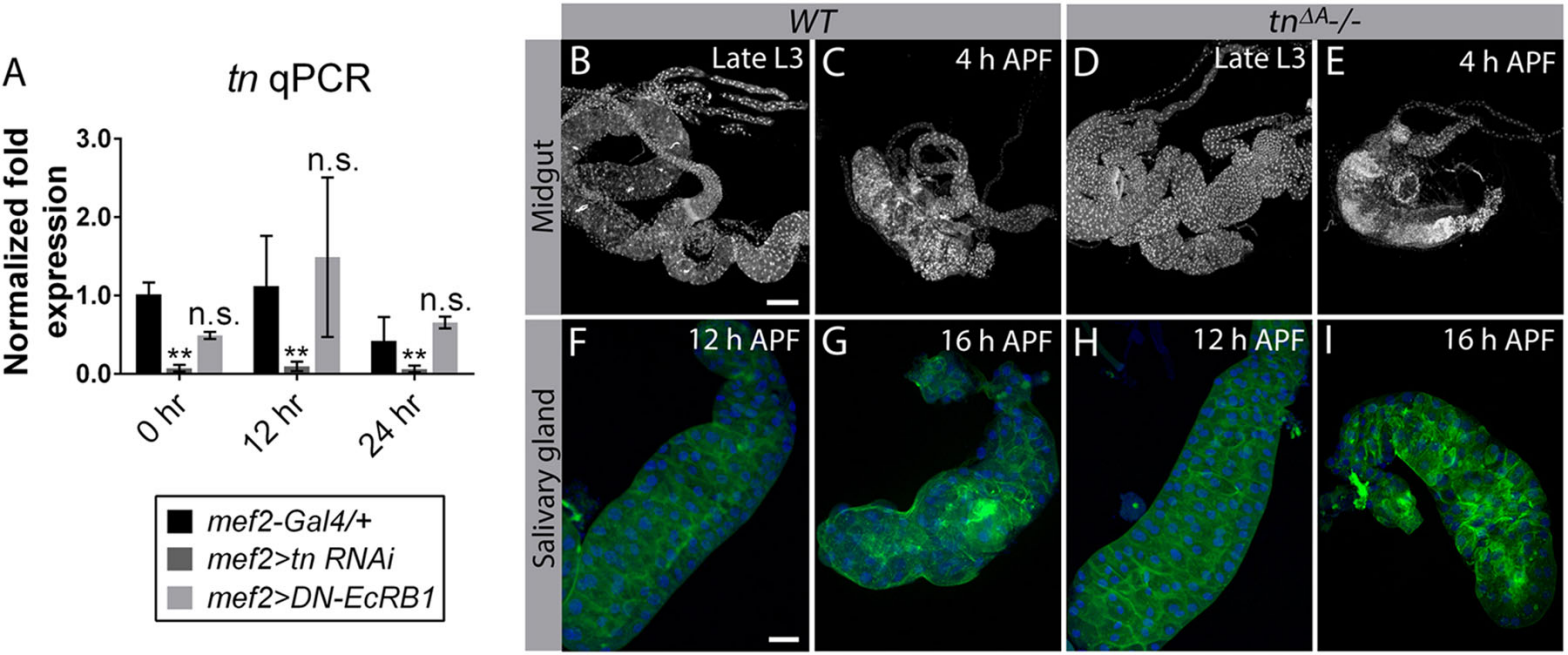
Tn is not an ecdysone target and is not required for general tissue histolysis. **a** *tn mRNA* levels measured by qPCR are not significantly different from controls (*mef2-Gal4/+*) when a dominant-negative version of isoform EcRB1 is expressed in muscle tissue during pupal morphogenesis. Note that *tn* transcript levels are decreased as expected upon muscle-specific *tn RNAi* knockdown. *N* = 3 biological replicates and 3 technical replicates for each genotype. **b**–**i** Z-stack confocal images of midguts (**b**–**e**) or salivary glands (**f**–**i**). Midgut histolysis proceeds normally by 4 h APF in *WT* (**b**, **c**) or *tn* mutants (**d**, **e**) as assayed by DAPI staining. PCD still occurs in salivary glands (F-actin, green and DAPI, blue) by 16 h APF upon loss of Tn (**h**, **i**) compared to *WT* controls (**f**, **g**). Mean ± SEM (n.s., not significant, **p *<* 0.01). Scale bars, 200 µm (**b**–**e**) and 100 µm (**f**–**i**)

As ecdysone signaling directs tissue histolysis during metamorphosis^9^, we sought to examine if Tn is an ecdysone target. *tn mRNA* levels were measured in pupae with blocked ecdysone signaling prior to (0 h APF), during (12 h APF), or after (24 h APF) completion of histolysis using quantitative PCR (qPCR). There was no significant difference in *tn* transcript levels between *mef2-Gal4/+* or *mef2>DN-EcRB1* samples at any stage of development (Fig. 2a). The reduction in *tn* transcript levels in *mef2>tn RNAi* muscles is consistent with previous results^49^, further demonstrating the specificity of inducing *tn RNAi* in pupal muscles and the sensitivity of our qPCR approach. Our data demonstrate that ecdysone does not globally regulate *tn* expression during metamorphosis.

To test if Tn plays a broader role in general tissue histolysis, we examined whether Tn regulates midgut or salivary gland breakdown. The midgut undergoes a drastic reduction in size between the late L3 and early pupal stages, whereas salivary gland histolysis takes place between 12 h APF and 16 h APF^50^. *WT* and *tn−/−* midguts appeared similar at 4 h APF (Fig. 2b–e) and there was no obvious delay or impairment in salivary gland histolysis in *tn* mutants compared to *WT* at 16 h APF (Fig. 2f–i). Therefore, unlike abdominal muscle, Tn does not play a broader role in general tissue breakdown during metamorphosis.

### Loss of Tn affects DIAP1 protein levels and Caspase-3 activity

**Fig. 3:**
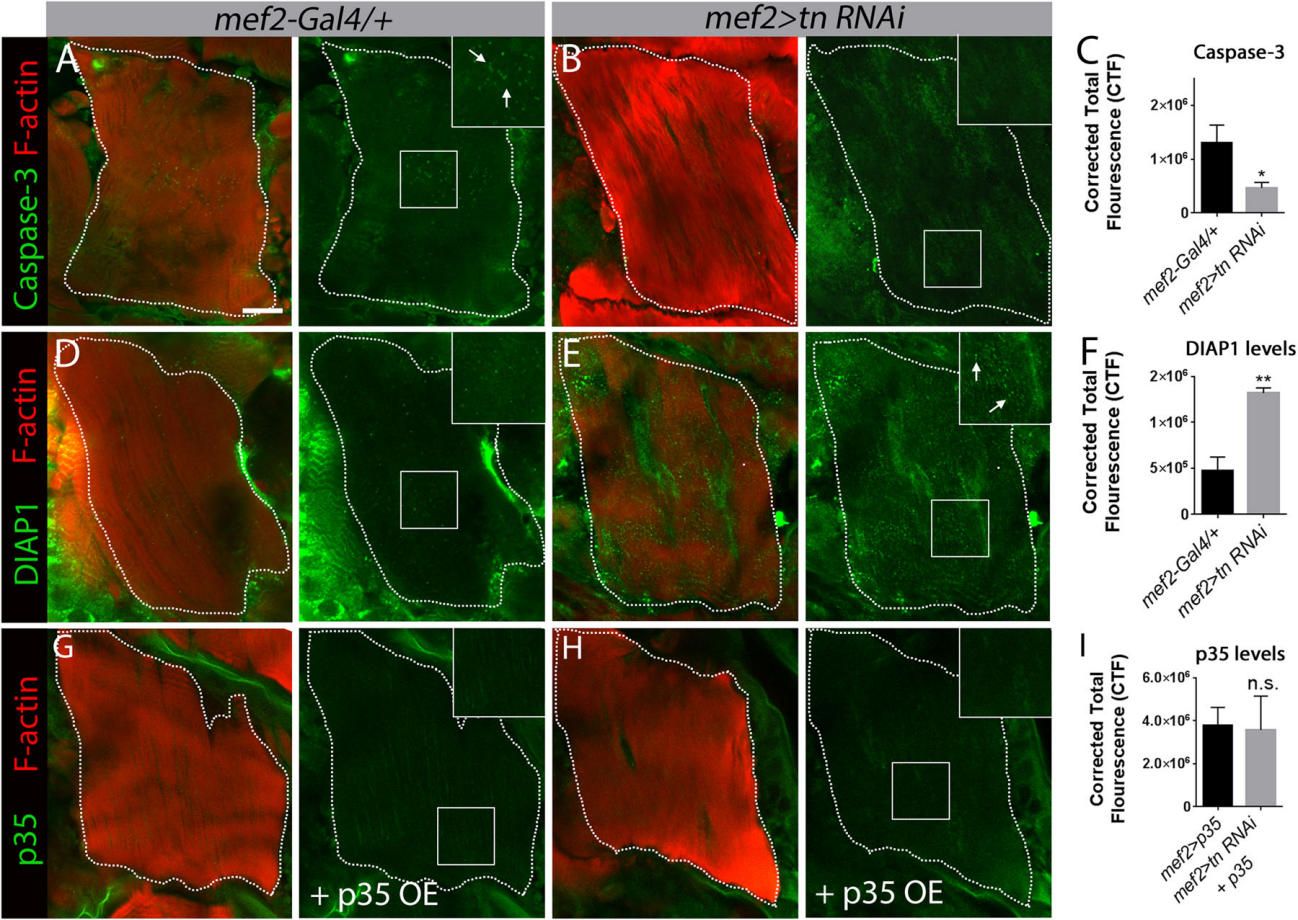
Tn affects DIAP1 and Caspase-3 activity during DEOM histolysis at 12 h APF. **a**–**c** In vivo labeling of Caspase-3 (green) in *mef2-Gal4/+* control or *mef2>tn RNAi* DEOMs co-labeled with phalloidin (red). **a** Puncta corresponding to Caspase-3 are present in degenerating DEOM controls (white arrows in inset). **b** No Caspase-3(+) puncta are present in *tn RNAi* muscles. **c** Quantification of the relative fluorescence intensities of Caspase-3 in DEOMs reflects the general decrease observed upon a reduction in Tn. **d**–**f** Immunostaining for DIAP1 (green) and F-actin (red) is higher upon disruption of Tn function (**e**, white arrows in inset) than in *mef2-Gal4/+* control muscles (**d**). **f** Bar graph showing that the relative DIAP1 fluorescence levels are increased upon reduced Tn function. **g**–**i** Overall p35 (green) levels in DEOMs labeled with F-actin (red) are not changed upon a reduction in Tn. *mef2>p35* appears similar when co-expressed in a *WT* (**g**) or *tn RNAi* (**h**) background. Quantification of fluorescence intensity reveals no significant difference between *WT* or experimental DEOMs (**i**). Mean ± SEM (n.s., not significant, **p < 0.01, *p *<* 0.05). Scale bar, 50 µm

Zirin et al.^28^ previously demonstrated that disintegration of the DEOMs relies on apoptosis. To understand if Tn facilitates DEOM cell death, we assayed protein levels of the initiator caspase Dronc since antibodies against cleaved-Caspase-3 are a read-out of Dronc activity^51^. Elevated Caspase-3 was present in *mef2-Gal4/+* muscles undergoing histolysis at 12 h APF (Fig. 3a, c). In contrast, there was an overall reduction in puncta corresponding to active Caspase-3 in muscles with disrupted Tn function (Fig. 3b, c). Next we examined DIAP1 protein levels. At 12 h APF, DIAP1 was present in DEOM controls at a basal level (Fig. 3d, f), whereas significantly higher DIAP1 levels were observed in *tn RNAi* DEOMs (Fig. 3e, f). This observed reduction in Dronc activity and the elevation of DIAP1 in *tn* mutants was not due to altered mRNA expression of *DIAP1*, *Dronc*, *Drice*, or *Dark* (Fig. S2). These data suggest that Tn post-transcriptionally regulates some of the cell death genes to direct muscle histolysis.

Since overexpression (OE) of the cell death inhibitor p35 blocks DEOM breakdown^28^, we next examined p35 to assess if Tn generally influences protein levels during histolysis or specifically functions in DIAP1-mediated apoptosis. The baculovirus p35 protein is not endogenously expressed in *Drosophila*, but inhibits effector caspases^52^. Thus, we used the Gal4/UAS system to target p35 in muscle alone (*mef2>p35*) or in a *tn RNAi* background (*mef2>tn RNAi+ p35 OE*). There was no significant difference in p35 levels in either genotype at 12 h APF (Fig. 3g–i). Moreover, there was no increase in the fluorescence intensity of DIAP1 upon p35 OE in the DEOMs (Fig. S3), proving that the observed accumulation of DIAP1 protein in *tn−/−* is not a secondary consequence of a block in muscle histolysis. Collectively, these findings show that Tn regulates DEOM cell death by influencing DIAP1 protein levels and Dronc activity.

### *tn* functions in a pathway with cell death genes

**Fig. 4:**
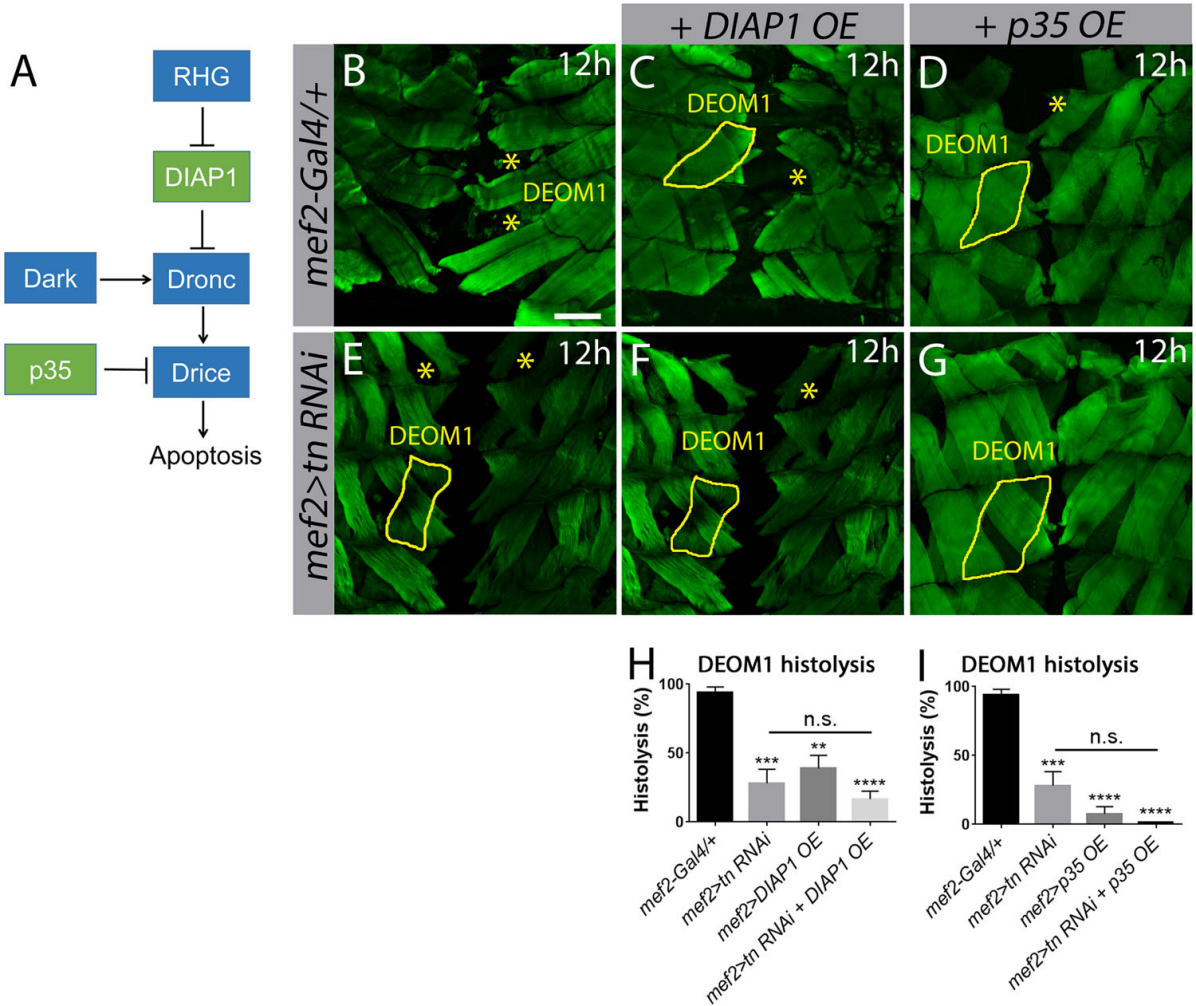
*tn* genetically interacts with *DIAP1* during DEOM histolysis. **a** Schematic of *Drosophila* core cell death machinery. **b**–**g** Merged Z-stack confocal images of the abdominal muscles stained with phalloidin (green) at 12 h APF. DEOM1 is outlined with a solid yellow line and histolysed DEOM1s are marked by yellow asterisks. **b** Histolysis proceeds normally in *mef2-Gal4/+* muscles. Overexpression of *DIAPI* (**c**) or *p35* (**d**) in muscles partially blocks DEOM1 histolysis. **e**–**g** There is no significant difference in DEOM1 histolysis upon RNAi knockdown of *tn* alone (**e**), or with overexpression of *DIAP1* (**f**) or p*35* (**g**) in a *tn RNAi* background. **h** A bar graph showing similar levels of muscle histolysis in *mef2>tn RNAi+DIAP1 OE* compared to *mef2>tn RNAi* muscles. **i** Quantification showing no significant difference in DEOM1 breakdown between *mef2>tn RNAi* and *mef2>tn RNAi+p35 OE* pupae. Mean ± SEM (n.s., not significant, ****p < 0.001, ***p < 0.005, **p < 0.01). Scale bar, 100 µm (**b**–**g**)

In the presence of cell death signals, the pro-apoptotic RGH proteins block DIAP1 inhibition, thereby enabling Dronc activation^2, 35^. Stable Dronc protein is activated by Dark and this Dronc–Dark apoptosome complex promotes the activity of effector caspases such as Drice for the execution of cell death (Fig. 4a)^44, 45^. Since our results show that *tn RNAi* blocks DEOM histolysis by altering DIAP1 and Caspase-3, we next sought to genetically manipulate cell death pathway components in a *tn RNAi* background to determine if Tn directly functions in apoptosis. Cell death was first blocked by expressing the cell death inhibitors *DIAP1* (*mef2>DIAP1 OE*) or p35 (*mef2>p35 OE*). By 12 h APF, all DEOM1s underwent histolysis in *mef2-Gal4/+* controls (Figs. 1i, k and 4b). Muscle-targeted DIAP1 OE inhibited degradation in approximately 50% of DEOM1s (Fig. 4c, h). In a *tn RNAi* background, DIAP1 OE did not enhance the extent of DEOM1 histolysis (Fig. 4f, h), suggesting that *tn* and *DIAP1* may be acting in concert to regulate muscle degradation. In contrast, exogenous expression of p35 was more effective in the prevention of DEOM1 degeneration alone (Fig. 4d, i) and in combination with *tn RNAi* (Fig. 4g, i), presumably due to the ability of p35 to block three different effector caspases (Drice, Dcp-1, and Decay)^3^. The addition of exogenous UAS elements (*UAS-GFP OE* or *UAS-GFP RNAi*) in the same *tn RNAi* background did not alter DEOM1 histolysis, indicating that sufficient Gal4 protein is present to drive all UAS-based constructs in the presence of *UAS-tn RNAi* (Fig. S1).

**Fig. 5:**
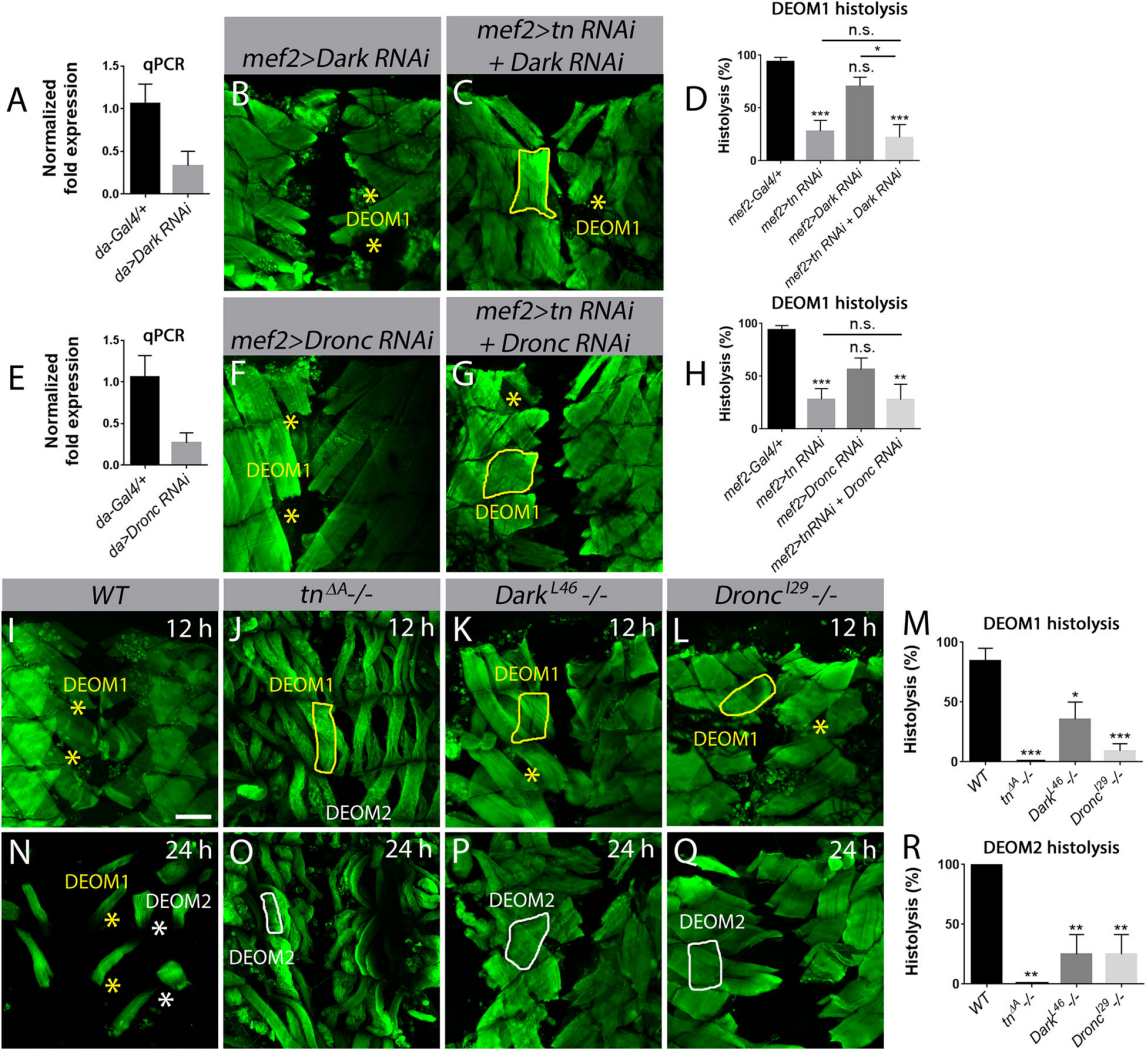
*Dronc* and *Dark* are required for DEOM histolysis. Merged Z-stack confocal images of the abdominal muscles stained with phalloidin (green) at 12 h APF (**b**, **c**, **f**, **g**, **i**–**l**) or 24 h APF (**n**–**q**). DEOM1s are outlined with a solid yellow line, DEOM2s with a solid white line. Histolyzed DEOM1 and DEOM2 muscles are marked by yellow and white asterisks, respectively. **a** *Dark* transcript levels are reduced by over 50% using the ubiquitous *da-Gal4* driver. *N* = 3 biological replicates and 3 technical replicates for each genotype. **b**, **c** DEOM1 histolysis is partially blocked upon RNAi knockdown of *Dark* alone (**b**) or if *Dark* is reduced in a *tn RNAi* background at 12 h APF. **d** Quantification of DEOM1 histolysis reveals a slight enhancement in muscle degeneration in *mef2>tn RNAi+Dark RNAi* compared to *mef2>Dark RNAi* alone. **e** Quantification of *Dronc* transcript levels show over a 60% decrease upon expression of *UAS-Dark RNAi* under control of *da-Gal4*. *N* = 3 biological replicates and 3 technical replicates for each genotype. **f**–**h** There is no significant difference in the histolysis of DEOM muscles in *mef2>tn RNAi+Dronc RNAi* pupae compared to *Dronc RNAi*. **i**–**l** *WT* DEOM1s have histolyzed by 12 h APF (**i**). There is a strong block in muscle breakdown in *tn*^*ΔA*^ (**j**), *Dark*^*L46*^ (**k**), and *Dronc*^*I29*^ (**l**) homozygous mutants at this same time point. **m** A bar graph showing the quantification of DEOM1 histolysis in (**i**–**l**). **n**–**q** All DEOM muscles are absent in *WT* pupae at 24 h APF (**n**). *tn*^*ΔA*^ (**o**), *Dark*^*L46*^*−/−* (**p**), and *Dronc*^*I29*^*−/−* (**q**) show significantly reduced DEOM2 breakdown at 24 h APF. **r** Quantification of DEOM2 histolysis in *tn*^*ΔA*^, *Dark*^*L46*^, and *Dronc*^*I29*^ mutants. Mean ± SEM (n.s., not significant, ***p < 0.005, **p < 0.01, *p < 0.05). Scale bar, 100 µm (**b**, **c**, **f**, **g**, **i**–**l**, **n**–**q**)

Next, we utilized muscle-specific RNAi to silence *Dronc* (*mef2>Dronc RNAi*) or *Dark* (*mef2*>*Dark RNAi*) to mimic a reduction in apoptotic signaling at 12 APF. In contrast to blocking apoptosis through OE of DIAP1 or p35, RNAi knockdown of *Dark* (Fig. 5b, d) or *Dronc* (Fig. 5f, h) alone was less effective at blocking DEOM1 histolysis. qPCR analysis revealed that *Dark* (Fig. 5a) and *Dronc* (Fig. 5e) transcript levels were decreased by ~60% under control of the ubiquitous *daughterless (da)-Gal4* promoter, likely accounting for the weaker block in histolysis than DIAP1 OE. The extent of muscle histolysis was not enhanced in a *tn RNAi* background upon a further reduction in *Dark* (Fig. 5c, d) or *Dronc* (Fig. 5g, h), further supporting the model that Tn acts with cell death genes to regulate DEOM histolysis.

Due to this partial block of Dark or Dronc caspase activity using RNAi, we next examined loss-of-function alleles. Both homozygous *Dark*^*L46*^ and *Dronc*^*I29*^ individuals are partially pupal lethal^53, 54^ but survive through muscle remodeling. At 12 APF, DEOM1 histolysis was blocked in both *Dark* (Fig. 5k, m) and *Dronc* (Fig. 5l, m) mutants, although to a lesser extent than *tn−/−*, where muscle degradation was completely abolished (Fig. 5j, m). At 24 h APF when DEOM1 and DEOM2 were normally absent in *WT* pupae (Fig. 5n, r), the majority of DEOM2s failed to undergo histolysis upon loss of Tn (Fig. 5o, r), Dark (Fig. 5p, r), or Dronc (Fig. 5q, r). Together, these results show that Tn mediates muscle breakdown by acting through the DIAP1-Dronc pathway.

### The RING domain of Tn is required for DEOM histolysis

**Fig. 6:**
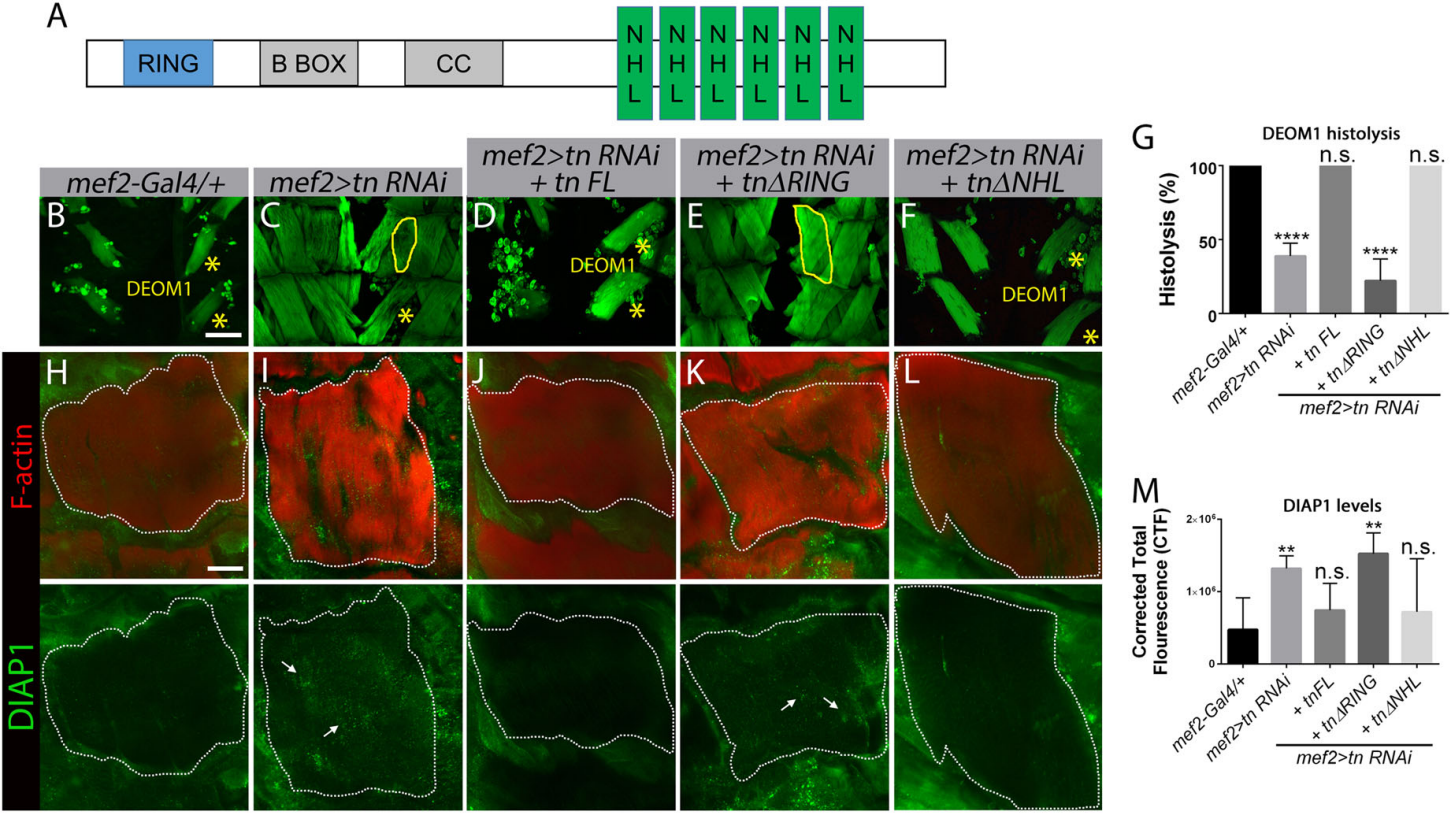
The RING domain is required for DEOM histolysis and to maintain DIAP1 protein levels. **a** Schematic diagram showing the conserved RING and NHL domains in Tn. The B-box and coiled-coil domains are predicted, but poorly conserved. **b**–**f** Confocal Z-stack micrographs of abdominal muscles at 24 h APF stained for F-actin (green). **b** DEOM1 underwent histolysis (*) in the *mef2-Gal4/+* control genotype. **c** DEOM1 histolysis is incomplete (yellow solid line) in *mef2>tn RNAi* animals. **d**–**f** Expression of Tn FL (**d**) or TnΔNHL (**f**) restores histolysis, while removal of the RING domain completely blocks DEOM degeneration, indicating this region is essential for normal histolysis (**e**). **g** Bar graph showing a significant decrease in DEOM histolysis in *mef2>tn RNAi; tnΔRING* flies. **h**–**l** Confocal Z-stack merged images of DEOM1 (white dotted line) at 12 h APF co-stained for F-actin (red) and DIAP1 (green). Low DIAP1 levels are observed in genotypes in which DEOM histolysis proceeds normally, including *mef2-Gal4* (**h**), *mef2>tn RNAi+tn FL* (**j**), and *mef2>tn RNAi+tnΔNHL* (**l**). Increased DIAP1 levels are observed in *mef2>tn RNAi* (**i**) or *mef2>tn RNAi+tnΔRING* (**k**) DEOMs. **m** Quantification of relative fluorescence intensity levels reveals significantly higher DIAP1 levels in *mef2>tn RNAi+tnΔRING* muscles. Mean ± SEM (n.s., not significant, ****p < 0.001, **p < 0.01). Scale bar, 100 µm (**b**–**f**) and 50 µm (**h**–**l**)

*Drosophila* Tn contains a conserved N-terminal RING domain and six NHL repeats in the C terminus (Fig. 6a). The B-Box and coiled-coiled (CC) regions are poorly conserved^48^. The requirement for the RING and NHL regions was investigated using genetic rescue experiments. We examined the effects of expressing full-length Tn (Tn FL) or versions of Tn lacking the RING (*tnΔRING*) or NHL domains (*tnΔNHL*) in DEOMs subjected to *tn RNAi* at 24 h APF. Transgene expression was confirmed by Tn immunostaining in the DEOMs and qPCR for *tn* transcript quantitation (Fig. S4). As expected, restoration of DEOM histolysis was observed upon the introduction of Tn FL in a *tn RNAi* background (Fig. 6d, g) compared to *tn RNAi* alone (Fig. 6c, g). DEOM1 was still intact at 24 h APF upon loss of the RING domain in Tn (Fig. 6e, g), while the presence of the RING domain (*mef>tn RNAi+tnΔNHL*) restored DEOM histolysis (Fig. 6f, g). These results indicate that the RING domain of Tn is required to prevent muscle breakdown.

The RING domain in E3 ubiquitin ligases such as Tn is required to transfer ubiquitin moieties from an E2 enzyme to a target substrate for proteasomal degradation^55^. Thus, to test if the RING domain of Tn may regulate DIAP1 protein levels through a similar mechanism, we examined DIAP1 immunostaining in DEOMs at 12 h APF again using truncated Tn constructs. Consistent with previous results in Fig. 3e, DIAP1 protein was elevated upon a reduction in Tn (Fig. 6i, m). Expression of Tn FL (Fig. 6j, m) or Tn lacking the NHL region (Fig. 6l, m) reduced DIAP1 levels similar to control muscles (Fig. 6h, m). In contrast, significantly higher DIAP1 was present in DEOMs expressing TnΔRING in a *tn RNAi* background (Fig. 6k, m). These results substantiate the importance of the RING domain in DEOM histolysis, specifically suggesting that DIAP1 is a substrate for Tn-mediated E3 activity.

### Tn acts via DIAP1 to regulate Dronc activity

**Fig. 7:**
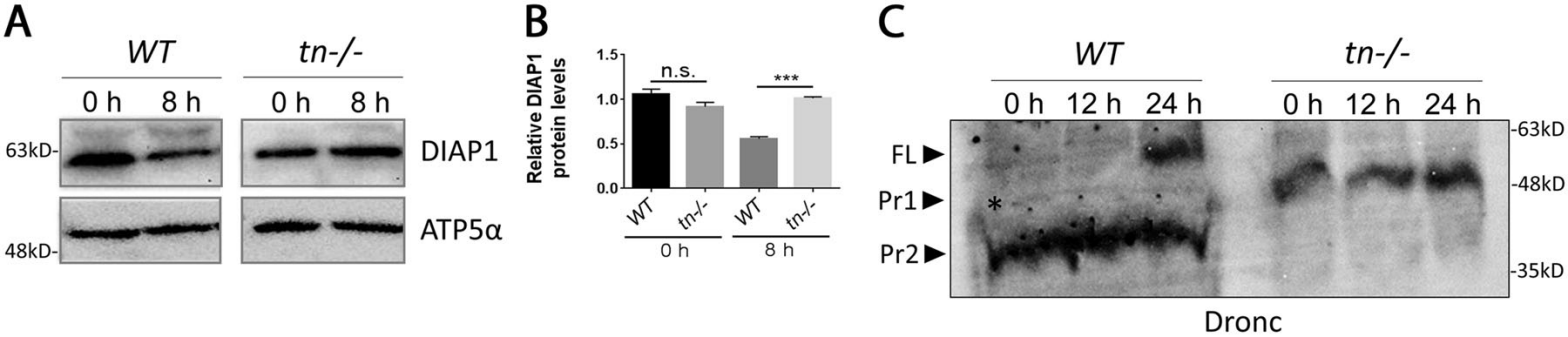
Loss of Tn affects DIAPI protein levels and Dronc activation. **a** Total protein extracted from pupae at 0 or 8 h APF was subjected to western blotting to assess DIAP1 or ATP5α protein levels. DIAP1 levels at 8 h APF are increased in *tn* mutants. **b** Densitometry quantification of the relative DIAP1 protein levels using ATP5α as a loading control reveals a significant increase in DIAP1 protein at 8 h upon loss of Tn (*n* = 3). **c** Dronc processing in *WT* or *tn−/−* pupae. Dronc FL (~55 kDa) is present at 24 h APF in *WT* samples. The cleaved Pr1 form (~40 kDa) is weakly observed during *WT* metamorphosis (black asterisk), while the active Pr2 cleavage product of Dronc (~37 kDa) predominates from 0 to 24 h APF. Only the Pr1 form is present upon loss of Tn throughout DEOM histolysis. Mean ± SEM (n.s., not significant, ***p < 0.005)

To further verify that loss of Tn alters DIAP1 levels, we performed western blots to quantify DIAP1 protein in WT or *tn−/−* pupae. Before the initiation of DEOM destruction (0 h APF), there was no significant difference in DIAP1 levels between WT or *tn* mutants (Fig. 7a, b). However, at 8 h APF, DIAP1 levels were approximately 2-fold higher upon loss of Tn. Since a primary role for DIAP1 is to bind and inhibit Dronc activity, we next examined Dronc processing. During apoptosis, FL Dronc is cleaved to produce Pr1 and Pr2 forms^56–58^. DIAP1 physically interacts with the FL and Pr1 forms of Dronc, thus preventing further cleavage to the active Pr2 protein. In *WT* pupae at 24 h APF, both the FL and processed Pr1 and Pr2 Dronc proteins were present (Fig. 7c). However, only the active form of Dronc was present from 0 h to 12 h APF, consistent with the normal timing of DEOM muscle histolysis. Interestingly, loss of Tn resulted in solely the Pr1 form (Fig. 7c). These data support a model whereby the upregulation of DIAP1 protein upon Tn deficiency prevents full Dronc activation, thus preventing apoptosis.

## Discussion

PCD is required for the destruction of certain larval tissues during metamorphosis^4, 9, 59^. Zirin et al.^28^ established that histolysis of the abdominal muscles is regulated by apoptosis, while blocking autophagy does not affect muscle breakdown^28^. In addition to the ecdysone receptor, only a handful of nuclear proteins are known to function in DEOM histolysis. Loss of East results in a partial block in DEOM degeneration, whereas premature muscle destruction is observed in muscles that lack Chromator^27^. Moreover, the two nuclear receptors, FTZ-F1 and HR39, antagonistically function to regulate the timing of DEOM histolysis^28^. Here we have further identified Tn as a novel protein in pupal muscle remodeling. However, loss of Tn does not affect salivary gland and midgut histolysis, highlighting an exclusive muscle role for Tn during *Drosophila* metamorphosis.

Our genetic assays demonstrate that *tn* functions with core components of the cell death machinery to regulate DEOM destruction. It was surprising that inhibition of apoptotic activity in *Dark* or *Dronc* mutants was not sufficient to completely block histolysis by 24 h APF. One explanation is the existence of additional cell death mechanisms other than apoptosis. While histolysing DEOMs contained autophagic vesicles, a reduction in autophagy components did not block or delay muscle degradation at 8 h APF^28^. We tested if a decrease in Tn-mediated apoptosis could sensitize muscle cells to initiate autophagy as a compensatory mechanism to assure cell death. However, this does not seem to be the case as RNAi knockdown of *Atg1*, *Atg5*, or *Atg18* does not further block DEOM histolysis in a *tn RNAi* background (Fig. S5). A second explanation is that the hypomorphic nature of these alleles may not completely abrogate Dark and Dronc function. Alternatively, additional effector caspases, including Dcp-1, Decay, and/or Damm, may be operating in the latter stages of DEOM histolysis since these caspases may function redundantly or act independent of the DIAP1–Drice axis^3^.

**Fig. 8:**
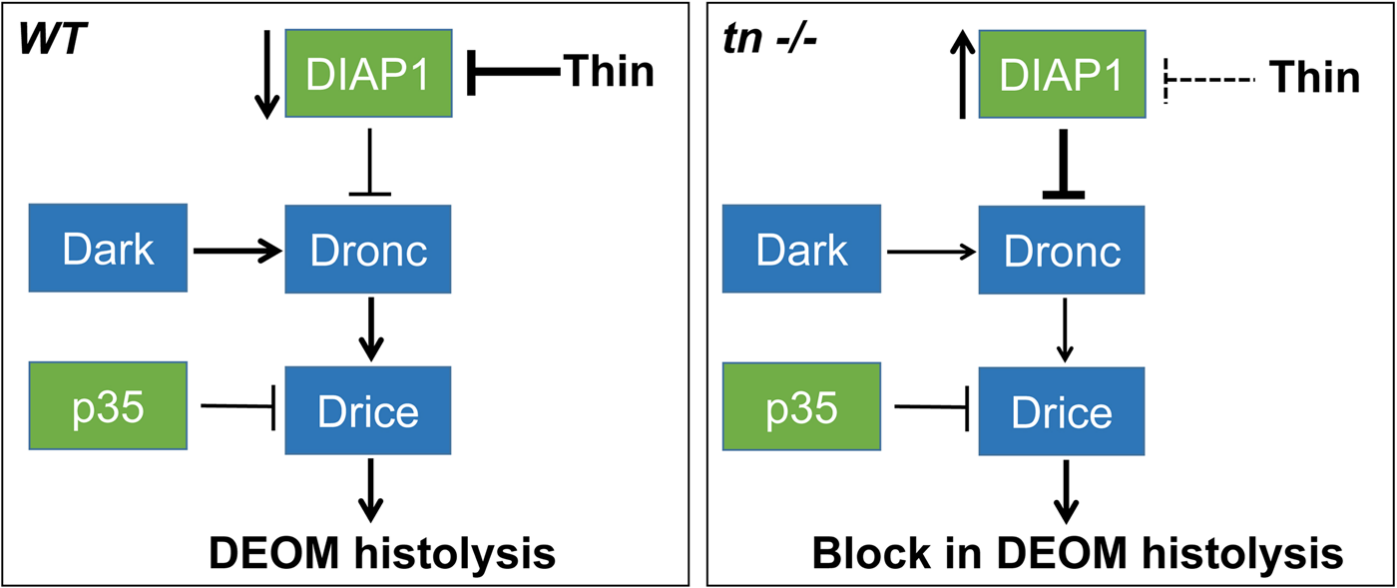
Model for Tn-mediated regulation of DEOM histolysis. **a** In *WT* DEOMs, Tn targets DIAPI for degradation. Downregulation of DIAP1 results in DEOM histolysis via caspase-dependent apoptosis. **b** In the absence of Tn, DIAPI levels increase and block caspase activity. Lack of caspase activation blocks cell death and preserves DEOMs

We expected more than a partial block in DEOM histolysis upon manipulation of DIAP1 (i.e., *DIAP1 OE* alone or *tn RNAi+DIAP1 OE*) at 12 h APF. It is possible that normal or overexpressed DIAP1 levels in the DEOMs are not high enough to block apoptosis, especially using RNAi approaches to reduce Tn levels. However, the use of *tn*-null alleles clearly shows a complete block in muscle degradation and a corresponding inhibition of active Dronc. Seemingly a delicate balance exists to regulate mRNA and protein expression, as well as protein turnover and proteolytic processing of active caspases. Cells must normally prevent cell death and only activate the apoptotic cascade upon a commitment to die. Thus, threshold levels of caspase activity must be reached for this terminal fate. There is evidence for stage or tissue-specific differential sensitivity to pro-apoptotic factors. Early L3 individuals are resistant to apoptosis, while wandering L3 larvae have elevated levels of Dark, Dronc, and Drice that are sufficient to trigger cell death under the appropriate stimuli^28^. We propose a model whereby Tn, through its RING domain, normally ubiquitinates DIAP1 for delivery to the proteasome during DEOM histolysis. This degradation of DIAP1 relieves Dronc inhibition, thereby initiating the caspase cascade for the execution of cell death (Fig. 8a). A general reduction in Tn, or loss of RING domain activity, prevents the addition of ubiquitin moieties and causes an increase in DIAP1 levels, effectively blocking cell death by limiting caspase activity (Fig. 8b).

Numerous roles have been identified for mammalian TRIM32 in normal and cancerous cells. In muscle, mutations in the NHL repeats result in limb-girdle muscular dystrophy type 2H or sarcotubular myopathy^60–66^. Several structural muscle proteins are targets of TRIM32 activity, including tropomyosin, desmin, α-actinin, and dysbindin^67–69^. However, it is not yet clear if regulation of these muscle substrates contributes to normal muscle physiology, is required to prevent atrophy, or plays a critical role in disease pathology^46^. The TRIM32-mediated degradation of additional protein substrates, including p53, Abi2, Piasy, and the X-linked IAP (XIAP), contribute to oncogenic or tumor suppressor activities that either confer resistance or susceptibility to apoptosis^70–73^. Tumor necrosis factor-α can trigger death receptor-mediated apoptosis through the regulation of XIAP activity^73^. TRIM32 colocalizes and directly interacts with XIAP in human kidney epithelial cells (HEK293). Moreover, TRIM32 induces apoptosis through the direct ubiquitination and subsequent protein turnover of XIAP degradation^73^. This control of apoptotic cell death mirrors our genetic results, strongly suggesting that this TRIM32-mediated regulation of IAP family members may be a conserved mechanism to regulate apoptosis. It would be interesting to further investigate if Tn and mammalian TRIM32 regulates apoptotic decisions in other contexts of muscle development and/or disease.

Herein, we have provided the first evidence for Tn in the regulation of muscle histolysis. Importantly, our genetic assays suggest that DIAP1 is a target of Tn and that regulation of DIAP1 and/or Caspase activity are crucial for a cell’s decision to execute cell death. These findings will further increase our general understanding about PCD during tissue destruction in *Drosophila* development and will provide a conserved framework to identify novel targets of Tn.

## Materials and methods

### Fly genetics

*Drosophila melanogaster* stocks were raised on standard cornmeal medium at 25 °C, unless otherwise indicated. The following fly stocks were used in this study: *w*^*1118*^ strain as *WT*; *mef2-Gal4* (Bloomington *Drosophila* Stock Center (BDSC), BL27390); *tn*^*ΔA*^ ^48^, two different *UAS-tn RNAi* lines (Vienna *Drosophila* Resource Center (VDRC), v19290 and v19291); *UAS-EcR.B1* (BL6469); *UAS-DIAP1.H* (BL6657); *UAS-Dronc RNAi* (BL32963)^74^; *UAS-Dark RNAi* (BL 33924); *UAS-p35.BH2* (BL5073); *UAS-tn FL*^48^. The *Dark*^*L46*^ and *Dronc*^*I29*^ alleles were generously provided by Bergmann and co-workers^53, 54^. The *mef2-Gal4; UAS-tn RNAi (mef2>tn RNAi)* line was created using standard recombination techniques and is maintained at 18 °C to maintain viability as 25 °C results in partial pupal lethality.

### Immunostaining and microscopy

To examine the effect of Tn in DEOM histolysis, white prepupae at 0 h were collected and aged until 8 h APF, 12 h APF, or 24 APF. Muscle preparations were dissected, fixed with 4% formaldehyde in phosphate-buffered saline (PBS) for 30 min and immunostained as indicated^75^. To analyze Tn function in salivary gland and midgut histolysis, wandering L3 larvae were dissected, fixed with 4% formaldehyde in PBS, and stained with DAPI (4′,6-diamidino-2-phenylindole) and/or phalloidin. The following primary antibodies were used: rabbit anti-Caspase-3 (1:100, Cell Signaling Technology, Danvers, MA,USA), mouse anti-DIAP1 (1:200, B. Hay)^76^, guinea pig anti-p35 (1:10, P. Meier)^40^, and guinea pig anti-Tn^48^. Secondary antibodies used for fluorescent immunolabeling were Alexa Fluor anti-mouse 488, Alexa Fluor anti-rabbit 488, Alexa Fluor anti-mouse 594, and Alexa Fluor anti-guinea pig 488 (1:400, Molecular Probes, Eugene, OR, USA). Phalloidin 488 and 594 were used for F-actin labeling (1:400, Molecular Probes, Eugene, OR, USA). Immunostained preparations were imaged on a Zeiss LSM 700, processed using the Zeiss Zen software and assembled into figures in Photoshop Elements.

### Western blotting

Five to ten pupae of the appropriate genotype were homogenized in 3× Laemmli buffer (150 mM Tris-HCl (pH 6.8), 300 mM dithiothreitol, 6% sodium dodecyl sulfate (SDS), 0.3% bromophenol blue, and 30% glycerol), boiled at 100 °C for 10 min, and centrifuged at 13,000 × *g* to remove cellular debris. The resulting proteins were separated by SDS-polyacrylamide gel electrophoresis (SDS-PAGE), transferred to polyvinyl difluoride membrane, and probed with mouse anti-DIAP1 (1:500, B. Hays) or guinea pig anti-Dronc (1:400, P. Meier). Horse radish peroxidase-conjugated secondary antibodies (1:5000, GE Healthcare, UK) were used to detect the primary antibodies. Protein bands were visualized using the ECL Plus Western Blotting Detection Kit (ThermoFisher, Waltham, MA, USA) and analyzed with the FluorChem M system (Protein Simple). The blots were stripped (6.25 ml of 1 M Tris-HCl, pH 6.8, 10 ml of 20% SDS, and 700 μl β-mercaptoethanol) and reprobed with mouse anti-ATP5α (1:10,000, Abcam, UK). Densitometry analysis was performed by calculating the band intensities of DIAP1 relative to the ATP5α loading control using Image J.

### Quantitative PCR

RNA was isolated from a pool of five pupae using the RNAeasy Mini Kit (Qiagen, Valencia, CA, USA) for each genotype. Three separate pools were used for each time point. Either *mef2-Gal4/+* (Fig. 2) or *w*^*1118*^ (Fig. 6) were used as a control. After elution, RNA concentrations were determined and single strand complementary DNA (cDNA) was generated from 100 ng of RNA using the SuperScript VILO cDNA Synthesis Kit (Invitrogen, Carlsbad, CA, USA). For the qPCR reactions, each cDNA sample was diluted to 1:50 and mixed with Power UP SYBR Green Master mix and the appropriate primers (Applied Biosystems, Foster City, CA, USA). *rp49* was used as the reference gene. Primers were synthesized by Integrated DNA Technologies (IDT): *rp49*: 5′F-GCCCAAGGGTATCGACAACA-3′, 5R′-GCGCTTGTTCGATCCGTAAC-3′; *Dark*: 5′F-AAGTACAATGTGAGCCGCCT-3′, 5′R-CCCAAGTCTTTCCCGATCCC-3′; *Dronc*: 5′F-AGTCGGCCGATATTGTGGAC-3′, 5′R-ACATAAGGGGTGAGTGCTCC-3′; *Drice*: 5′F-GACTGCCGCTACAAGGACAT-3′, 5′R-TGATTGGCCGTGAAGAAGCT-3′; *Diap1*: 5′F-GCGTGGAAATCGGTTGCTG-3′, 5′R-GATGCGATCTAATGCTTCGGC-3′; *tn*: 5′F-GAGCTGCATATCGAAATCACCG-3′; 5′R-AGATAGGCTTTTTCCGAGCAAAC-3′.

Three independent biological replicates were processed for each genotype and reactions were run in triplicate using the Quant Studio 3 Applied Biosystem with Quant studio design and analysis software. The average of the triplicates was used to calculate the 2^−ΔΔCt^ values (normalized fold expression). Quantification of mRNA levels between different genotypes at the same time point was performed using multiple *t* tests. Two-way analysis of variance (ANOVA) was used to compare transcript levels of the same genotype at different developmental time points.

### Transgenic fly lines

PCR amplification from the *tn* cDNA GH06739 (*Drosophila* Genomic Resource Center) was used to generate *UAS-tn FL*, *UAS-tnΔRING*, and *UAS-tnΔNHL* using the following primers: UAS-Tn FL—5′F-TAACGCGTCGACAATGGAGCAATTCGAGCAGCTGTTGAC-3′, 5′R-CTAGTCTAGATCAGAAGACTTGGACGCGGTGATTC 3′; UAS-Tn ΔRING—5′F-AATAAGAATAGCGGCCGCATGAATCTGCGACGAGACATCACG-3′, 5′R- CTAGTCTAGATCAGAAGACTTGGACGCGGTGATTC 3′; UAS-TnΔNHL—5′F-TAACGCGTCGACAATGGAGCAATTCGAGCAGCTGTTGAC 3′. 5′R-CTAGTCTACATGCTGGCGCTTGCGCAGGTACACCTG 3′.

Each of the amplified regions was digested with *Sal*I/*Xba*I or *Not*I/*Xba*I (restriction sites underlined), subcloned into the pUAST *Drosophila* transformation vector, and verified by sequencing. Transgenic fly lines were generated by Genetic Services, Inc.

### Quantitative image analysis

*Initiation of muscle histolysis (%)*: The number of DEOM1 muscles that were morphologically smaller or fragmented (indicative of histolysis initiation) in abdominal segments A2 and A3 in control or experimental genotypes at 0 or 8 h APF was quantitated. The percent of muscle histolysis initiation is illustrated as a percent of smaller or fragmented DEOM1 muscles/total number of DEOM1 muscles analyzed.

*Histolysis (%)*: The extent of muscle histolysis was determined by counting the number of DEOM1 muscles present in abdominal segments A2 and A3 in control and experimental samples at the indicated time points (12 or 24 h APF). All values are portrayed in percentages as DEOM1 muscles absent/total number of DEOM1 muscles analyzed.

*Corrected total fluorescence (CTF)*: To measure the fluorescence intensities of Caspase-3, DIAP1, and p35 protein levels in dying DEOM1 or DEOM2 muscles, the CTF method was used^77, 78^. The net average fluorescence intensity in a region of interest was measured in single section planes (1 µm slices) inside the DEOMs and in an area without fluorescence for background subtraction. All measurements were performed in Image J.

*Statistical analysis*: All raw data were imported into GraphPad Prism 6.0 for statistical analysis and graph production. All error bars represent mean ± standard error of the mean (SEM). Statistical significances were determined using either a Student's *t* test, Mann–Whitney tests, or one-way ANOVA. Differences were considered significant if *p* < 0.05 and are indicated in each figure legend. All “*n*” values are listed in Supplementary Tables.

## Electronic supplementary material

Supplemental Material

Supplemental Material
