# Supplementary material for: Thin is required for cell death in the Drosophila abdominal muscles by targeting DIAP1
Source: Cell Death Dis. 2018 Jul 3;9(7):740. doi: 10.1038/s41419-018-0756-x (PMC6030163; doi:10.1038/s41419-018-0756-x)
Supplement: Supplementary file 1 — Supplemental Material [file 41419_2018_756_MOESM1_ESM.docx]

**Vishal, et al., FIGURE S1**

**
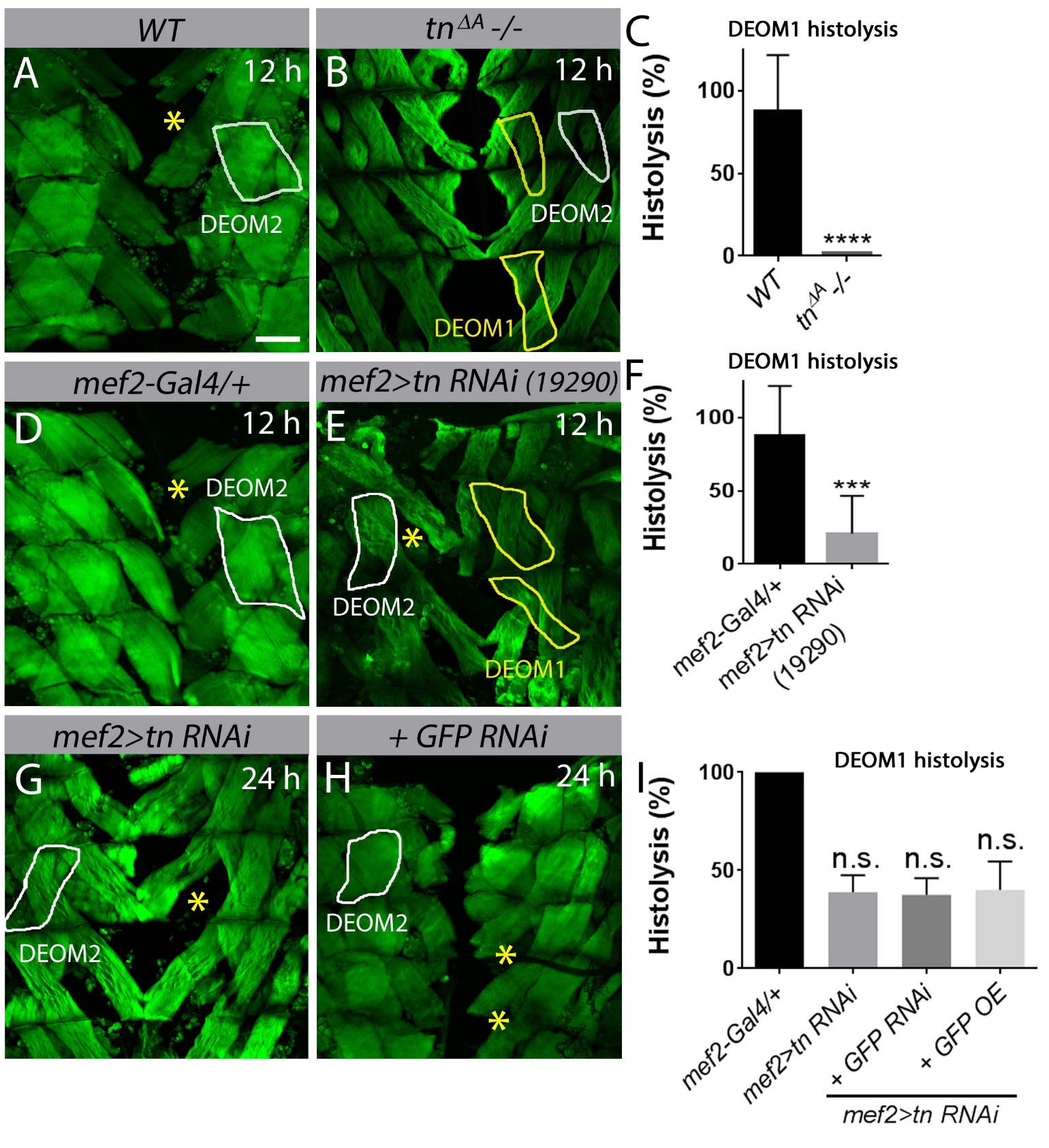
**

**Figure S1. Independent assessment of Tn function in the prevention of DEOM1 muscle breakdown.** (A-C) DEOM1 histolysis is blocked upon a complete loss of Tn at 12 h APF. (A) Histolysis is complete in *WT* DEOM1 muscles (yellow asterisk), while DEOM2 is still present at 12 h APF (white solid line) (B) All DEOM1 (yellow solid line) and DEOM 2 muscles have failed to degenerate in *tn* mutants. (C) Bar graph indicates the absence of DEOM1 histolysis in *tn -/-*. (D-F) Reduction of Tn with a second *tn RNAi* line (VDRC19290) shows a significant reduction in DEOM1 histolysis at 12 h APF. *mef2>tn RNAi* partially blocks DEOM1 muscle histolysis (E) compared to *mef2-Gal4/+* control muscles (D). (F) Quantification of DEOM1 histolysis in control or *tn RNAi* muscles. (G-I) The addition of an exogenous *UAS-GFP RNAi* (H) in a *mef2>tn RNAi* background (G) does not alter Tn-mediated muscle histolysis at 24 h APF. (I) The percentage of DEOM1 muscle histolysis in *tn RNAi* is comparable to the addition of GFP OE (*UAS-GFP*) or GFP RNAi *(UAS-GFP RNAi*). Thus an additional UAS element does not titrate out the amount of Gal4 protein used to drive *tn RNAi*. Mean ± SEM (n.s., not significant, **** p < 0.001, *** p < 0.005). Scale bar, 100µm.

**Vishal, et al., FIGURE S2**

**
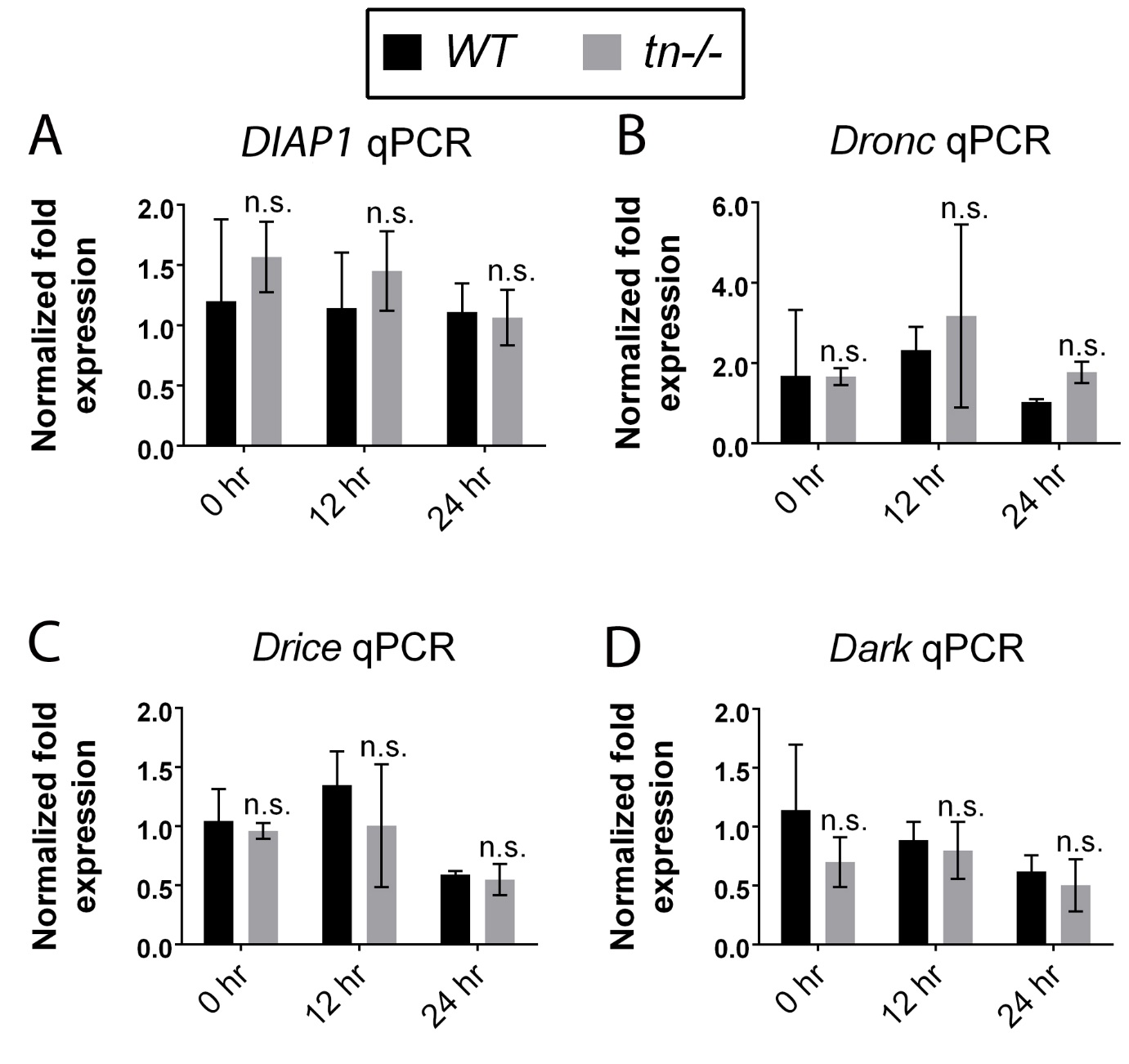
**

**Figure S2. *tn* does not regulate the transcript levels of cell death components.** (A-D) qPCR was used to assess mRNA levels from whole pupae in *WT* or *tn* mutants at the indicated time points before (0 h), during (12 h), or after (24 h) DEOM1 histolysis. There are no significant differences in the mRNA levels of *Diap1* (A), *Dronc* (B), *Dark* (C) or *Drice* (D) in *tn* -/- as compared to *WT* pupae at any stage of DEOM histolysis. N = 3 biological replicates and 3 technical replicates for each genotype. Mean ± SEM, (n.s., not significant).

**Vishal, et al., FIGURE S3**


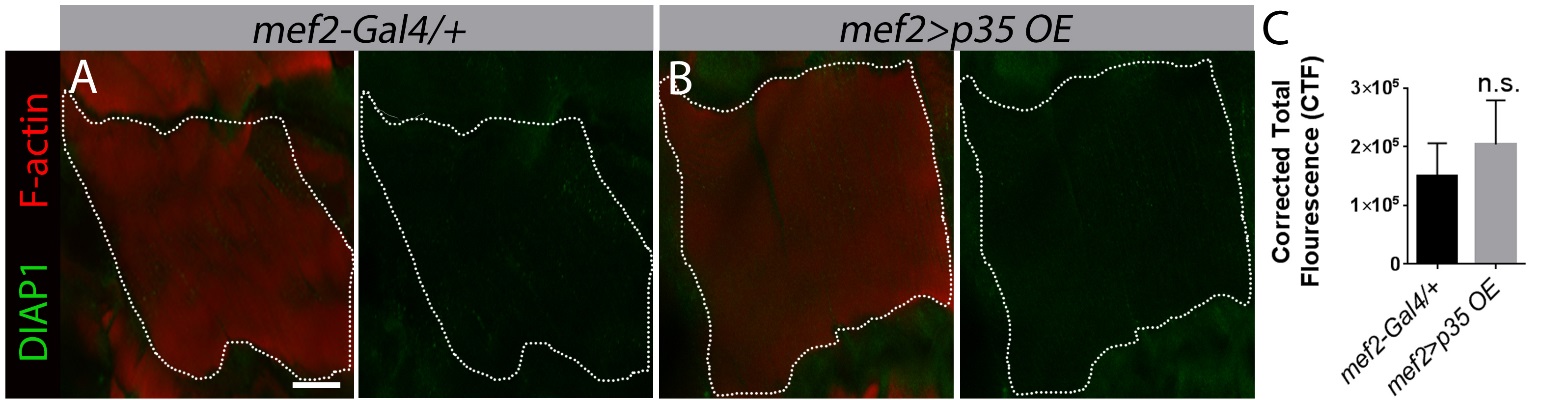


**Figure S3. Overexpressing *p35* does not affect DIAP1 levels in the DEOMs.** Confocal micrographs of abdominal muscles co-labeled for F-actin (red) and DIAP1 (green). (A-B) There is no significant difference in DIAP1 levels between control *mef2-Gal4/+* (A) or *mef2>p35 OE* (B) muscles at 12 h APF. (C) A bar graph showing the corrected total fluorescence of DIAP1 immunostaining in the indicated genotypes. Mean ± SEM, (n.s., not significant). Scale bar, 50µm.

**Vishal, et al., FIGURE S4**

**
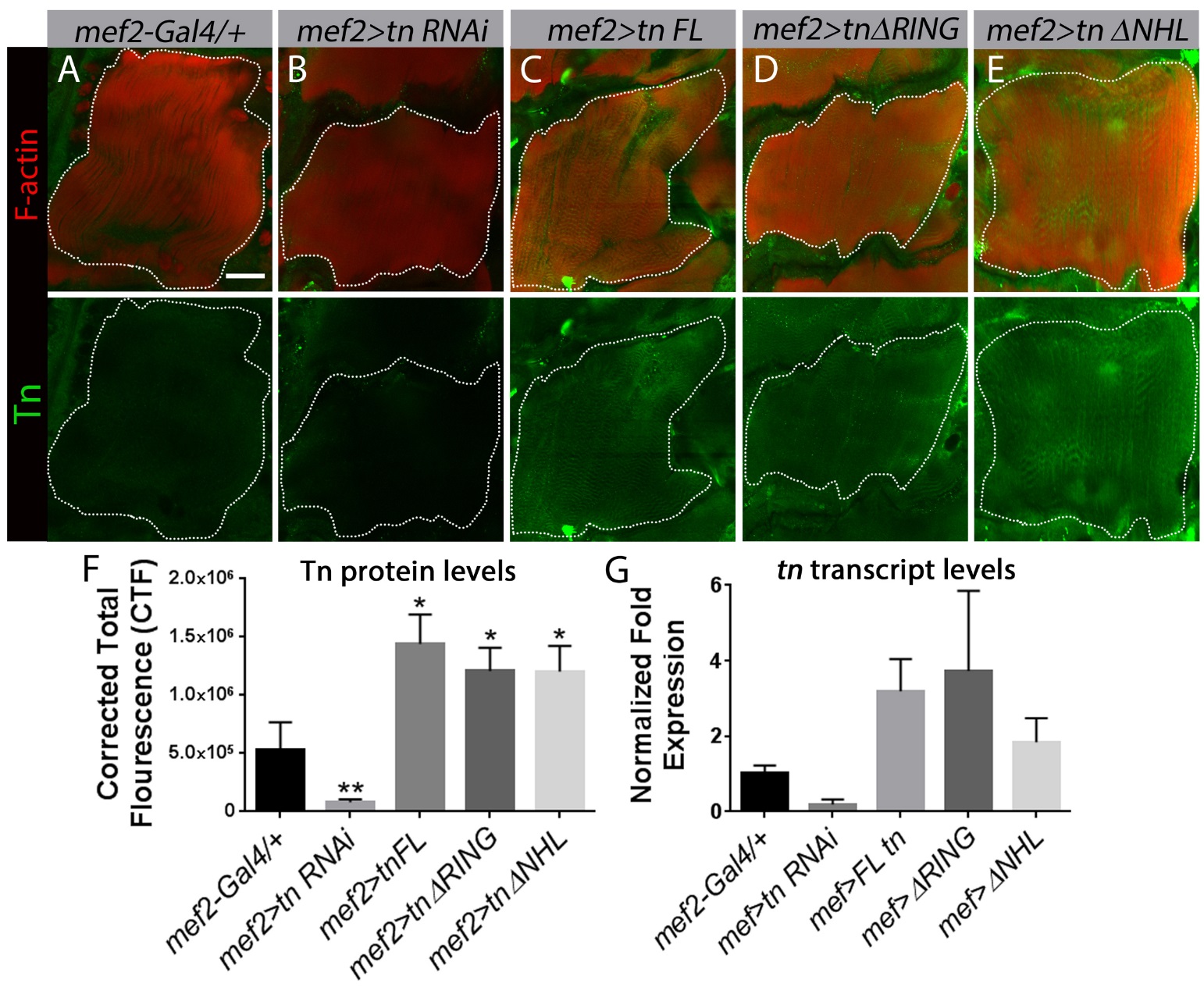
**

**Figure S4. Expression levels of Tn protein and *tn* transcripts at 12 h APF.** (A-E) Confocal micrographs of DEOMs to visualize F-actin (red) and Tn protein (green). (F) There is a significant upregulation of Tn protein levels in *mef2>tn FL, mef2>tnΔRING* and *mef2>tnΔNHL* DEOM muscles over *mef2-Gal4/+* control muscles or upon knockdown of *tn* using RNAi. (G) A bar graph showing upregulation of *tn* transcript levels in the indicated *UAS*-*tn* overexpression constructs. N = 3 biological replicates and 3 technical replicates for each genotype. Mean ± SEM, (**, p<0.01, * p<0.05). Scale bar, 50µm.

**Vishal, et al., FIGURE S5**


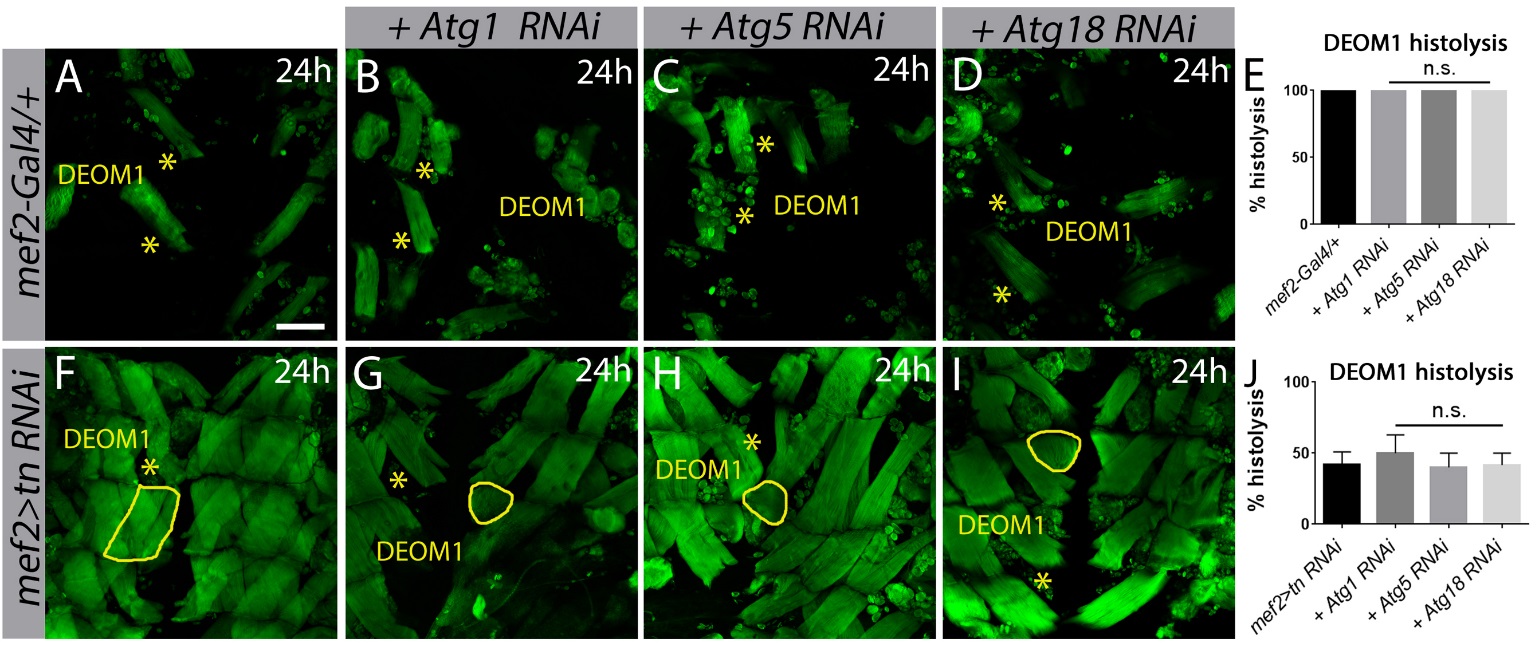


**Figure S5. DEOM histolysis does not require autophagy.** (A-D) There is no significant difference in DEOM1 breakdown between *mef2-Gal4/+* control (A) or *mef2>Atg1RNAi* (B), *mef2>Atg5 RNAi* (C) and *mef2>Atg 18a RNAi* (D) pupae. (E) A bar graph showing completet histolysis in all genotypes examined at 24 h APF. (F-I) DEOM degeneration is not significantly enhanced in upon a reduction in *Atg1* (G), *Atg5* (H), and *Atg18* (I) compared to *mef2;tn RNAi* alone (F). (J) Quantification of DEOM1 histolysis in (F-I). Mean ± SEM, (n.s., not significant). Scale bar, 100µm.

**Vishal, et al., FIGURE S6**

**
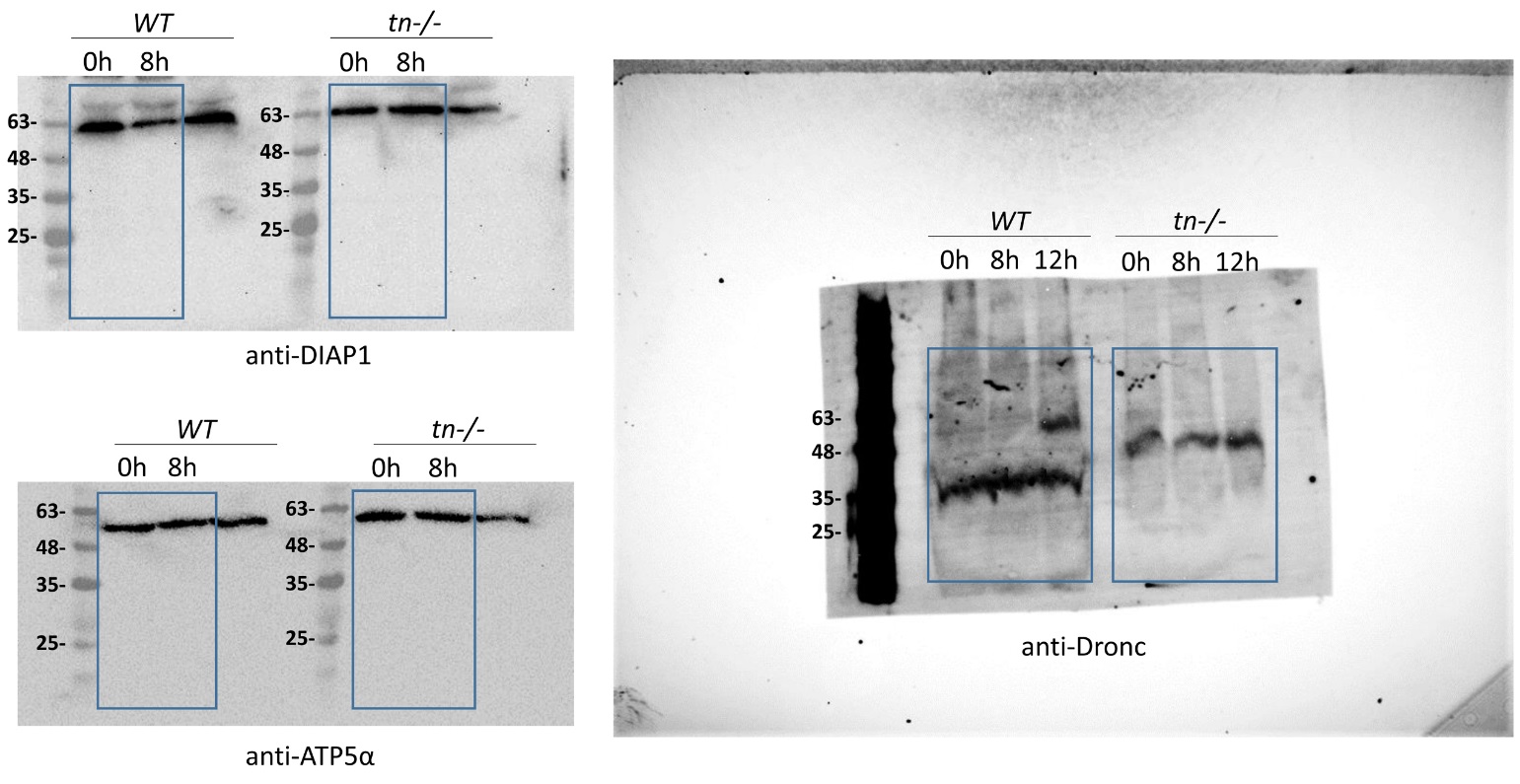
**

**Figure S6. Raw Western blot data for Figure 7.** Raw images shown here were cropped in Photoshop Elements and assembled as shown in Figure 7.

**Vishal, et al., TABLE S1**

| **Figure** | **Panel** | **Genotype** | **Description** | **N** |
| --- | --- | --- | --- | --- |
| Figure 1 | 1E | *mef2-Gal4/+* | 0h APF histolysis | 9 |
|  | 1H | *mef2-Gal4/+* | 8h APF histolysis | 8 |
|  | 1K | *mef2-Gal4/+* | 12h APF histolysis | 16 |
|  | 1N | *mef2-Gal4/+* | 24h APF histolysis | 16 |
|  | 1E | *mef2>tn RNAi* | 0h APF histolysis | 9 |
|  | 1H | *mef2>tn RNAi* | 8h APF histolysis | 10 |
|  | 1K | *mef2>tn RNAi* | 12h APF histolysis | 16 |
|  | 1N | *mef2>tn RNAi* | 24h APF histolysis | 19 |
| Figure 3 | 3C | *mef2-Gal4/+* | Caspase-3 | 7 |
|  | 3F | *mef2-Gal4/+* | DIAP1 | 9 |
|  | 3I | *mef2-Gal4/+* | p35 | 8 |
|  | 3C | *mef2>tn RNAi* | Caspase-3 | 9 |
|  | 3F | *mef2>tn RNAi* | DIAP1 | 10 |
|  | 3I | *mef2>tn RNAi* | p35 | 7 |
| Figure 4 | 4H | *mef2-Gal4/+* | 12h APF histolysis | 16 |
|  | 4H | *mef2>tn RNAi* | 12h APF histolysis | 16 |
|  | 4H | *mef>DIAP1 OE* | 12h APF histolysis | 18 |
|  | 4H | *mef2>tn RNAi + DIAP1 OE* | 12h APF histolysis | 18 |
|  | 4I | *mef2-Gal4/+* | 12h APF histolysis | 16 |
|  | 4I | *mef2>tn RNAi* | 12h APF histolysis | 16 |
|  | 4I | *mef>p35 OE* | 12h APF histolysis | 13 |
|  | 4I | *mef2>tn RNAi + p35 OE* | 12h APF histolysis | 11 |
| Figure 5 | 5D | *mef2-Gal4/+* | 12h APF histolysis | 16 |
|  | 5D | *mef2>tn RNAi* | 12h APF histolysis | 16 |
|  | 5D | *mef>Dark RNAi* | 12h APF histolysis | 17 |
|  | 5D | *mef2>tn RNAi + Dark RNAi* | 12h APF histolysis | 9 |
|  | 5H | *mef2-Gal4/+* | 12h APF histolysis | 16 |
|  | 5H | *mef2>tn RNAi* | 12h APF histolysis | 16 |
|  | 5H | *mef>DroncRNAi* | 12h APF histolysis | 16 |
|  | 5H | *mef2>tn RNAi + Dronc RNAi* | 12h APF histolysis | 9 |
|  | 5M | *WT* | 12h APF histolysis | 13 |
|  | 5M | *tn^ΔA^-/-* | 12h APF histolysis | 8 |
|  | 5M | *Dark^L46-^/-* | 12h APF histolysis | 7 |
|  | 5M | *Dronc^I29^-/-* | 12h APF histolysis | 11 |
|  | 5R | *WT* | 24h APF histolysis | 6 |
|  | 5R | *tn^ΔA^-/-* | 24h APF histolysis | 5 |
|  | 5R | *Dark^L46-^/-* | 24h APF histolysis | 8 |
|  | 5R | *Dronc^I29^-/-* | 24h APF histolysis | 8 |
| Figure 6 | 6G | *mef2-Gal4/+* | 12h APF histolysis | 16 |
|  | 6G | *mef2>tn RNAi* | 12h APF histolysis | 18 |
|  | 6G | *mef2>tn RNAi + tn FL* | 12h APF histolysis | 8 |
|  | 6G | *mef2>tn RNAi + tn*Δ*RING* | 12h APF histolysis | 9 |
|  | 6G | *mef2>tn RNAi + tnΔNHL* | 12h APF histolysis | 10 |
|  | 6M | *mef2-Gal4/+* | DIAP1 | 9 |
|  | 6M | *mef2>tn RNAi* | DIAP1 | 10 |
|  | 6M | *mef2>tn RNAi + tn FL* | DIAP1 | 8 |
|  | 6M | *mef2>tn RNAi + tn*Δ*RING* | DIAP1 | 7 |
|  | 6M | *mef2>tn RNAi + tnΔNHL* | DIAP1 | 7 |

**Table S1. ‘n’ for all graphs in manuscript figures.**

**Vishal, et al., TABLE S2**

| **Figure** | **Panel** | **Genotype** | **Description** | **N** |
| --- | --- | --- | --- | --- |
| Figure S1 | 1C | *WT* | 12h APF histolysis | 16 |
|  |  | *tn^ΔA^-/-* | 12h APF histolysis | 8 |
|  | 1F | *WT* | 12h APF histolysis | 16 |
|  |  | *mef2>tn RNAi (19290)* | 12h APF histolysis | 12 |
|  | 1I | *mef2-Gal4/+* | 24h APF histolysis | 16 |
|  |  | *mef2>tn RNAi* | 24h APF histolysis | 16 |
|  |  | *mef2>tn RNAi + GFP RNAi* | 24h APF histolysis | 16 |
|  |  | *mef2>tn RNAi + GFP OE* | 24h APF histolysis | 10 |
| Figure S3 | 3C | *mef2-Gal4/+* | DIAP1 | 6 |
|  |  | *mef2>p35 OE* | DIAP1 | 7 |
| Figure S4 | 4F | *mef2-Gal4/+* | Tn | 6 |
|  |  | *mef2>tn RNAi* | Tn | 8 |
|  |  | *mef2>tn RNAi + tn FL* | Tn | 8 |
|  |  | *mef2>tn RNAi + tn*Δ*RING* | Tn | 8 |
|  |  | *mef2>tn RNAi + tnΔNHL* | Tn | 6 |
| Figure S5 | 5E | *mef2-Gal4/+* | 24h APF histolysis | 6 |
|  |  | *mef2>Atg1 RNAi* | 24h APF histolysis | 5 |
|  |  | *mef2>Atg5 RNAi* | 24h APF histolysis | 6 |
|  |  | *mef2>Atg18 RNAi* | 24h APF histolysis | 6 |
|  | 5J | *mef2>tn RNAi* | 24h APF histolysis | 16 |
|  |  | *mef2>tn RNAi + Atg1 RNAi* | 24h APF histolysis | 6 |
|  |  | *mef2>tn RNAi + Atg5 RNAi* | 24h APF histolysis | 5 |
|  |  | *mef2>tn RNAi + Atg18 RNAi* | 24h APF histolysis | 6 |

**Table S2. ‘n’ for all graphs in supplemetal figures.**
