# Supplementary material for: Thin is required for cell death in the Drosophila abdominal muscles by targeting DIAP1
Source: Cell Death Dis. 2018 Jul 3;9(7):740. doi: 10.1038/s41419-018-0756-x (PMC6030163; doi:10.1038/s41419-018-0756-x)
Supplement: Supplementary file 2 — Supplemental Material [file 41419_2018_756_MOESM2_ESM.pdf]

Vishal, et al., FIGURE S1

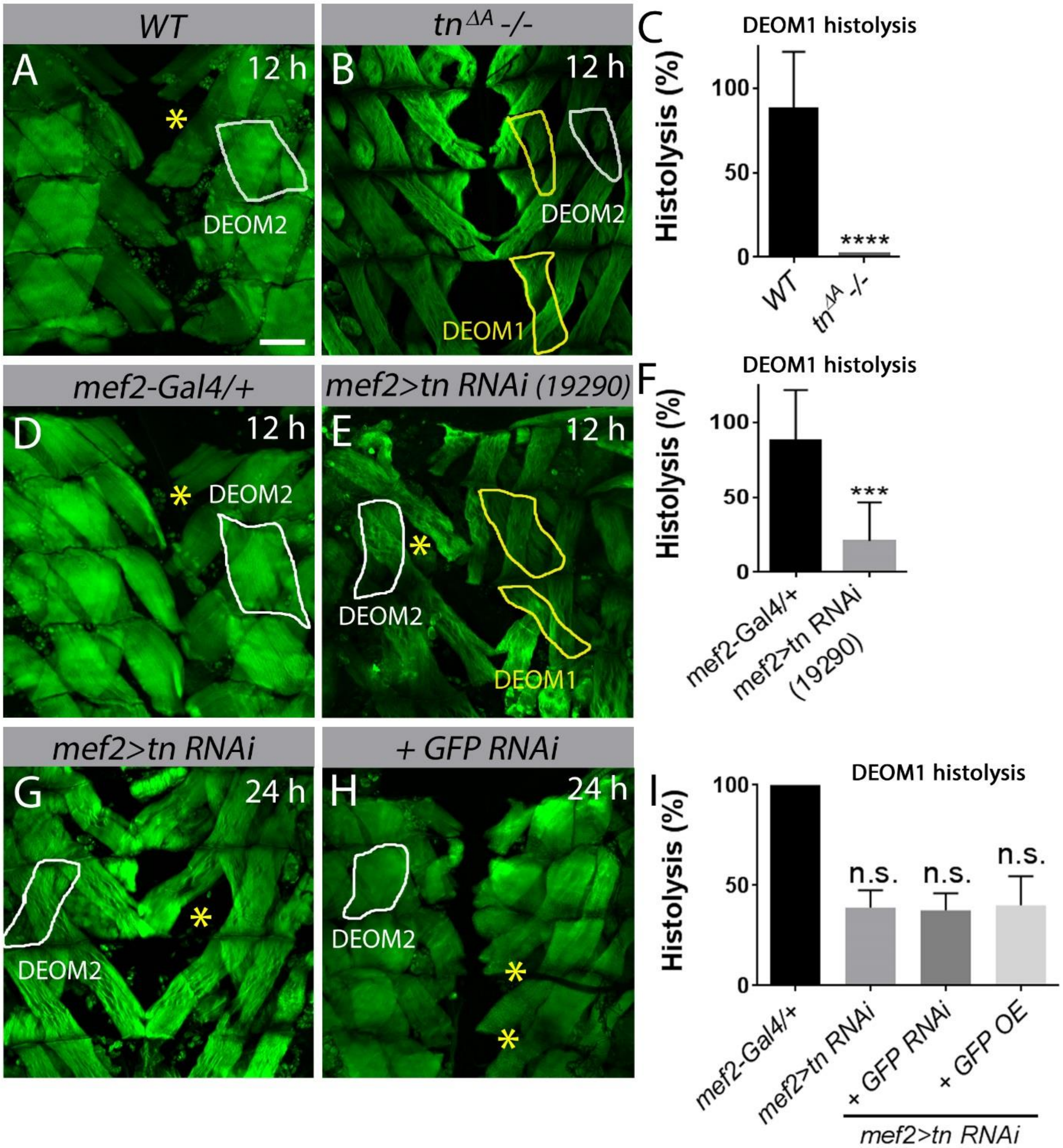

Figure S1. Independent assessment of Tn function in the prevention of DEOM1 muscle breakdown. (A-C) DEOM1 histolysis is blocked upon a complete loss of Tn at 12 h APF. (A) Histolysis is complete in *WT* DEOM1 muscles (yellow asterisk), while DEOM2 is still present at 12 h APF (white solid line) (B) All DEOM1 (yellow solid line) and DEOM 2 muscles have failed to degenerate in *tn* mutants. (C) Bar

graph indicates the absence of DEOM1 histolysis in *tn -/-*. (D-F) Reduction of Tn with a second *tn RNAi* line (VDRC19290) shows a significant reduction in DEOM1 histolysis at 12 h APF. *mef2>tn RNAi* partially blocks DEOM1 muscle histolysis (E) compared to *mef2-Gal4/+* control muscles (D). (F) Quantification of DEOM1 histolysis in control or *tn RNAi* muscles. (G-I) The addition of an exogenous *UAS-GFP RNAi* (H) in a *mef2>tn RNAi* background (G) does not alter Tn-mediated muscle histolysis at 24 h APF. (I) The percentage of DEOM1 muscle histolysis in *tn RNAi* is comparable to the addition of GFP OE (*UAS-GFP*) or GFP RNAi *(UAS-GFP RNAi*). Thus an additional UAS element does not titrate out the amount of Gal4 protein used to drive *tn RNAi*. Mean ± SEM (n.s., not significant, **** $p < 0.001$, *** $p < 0.005$). Scale bar, 100µm.

Vishal, et al., FIGURE S2

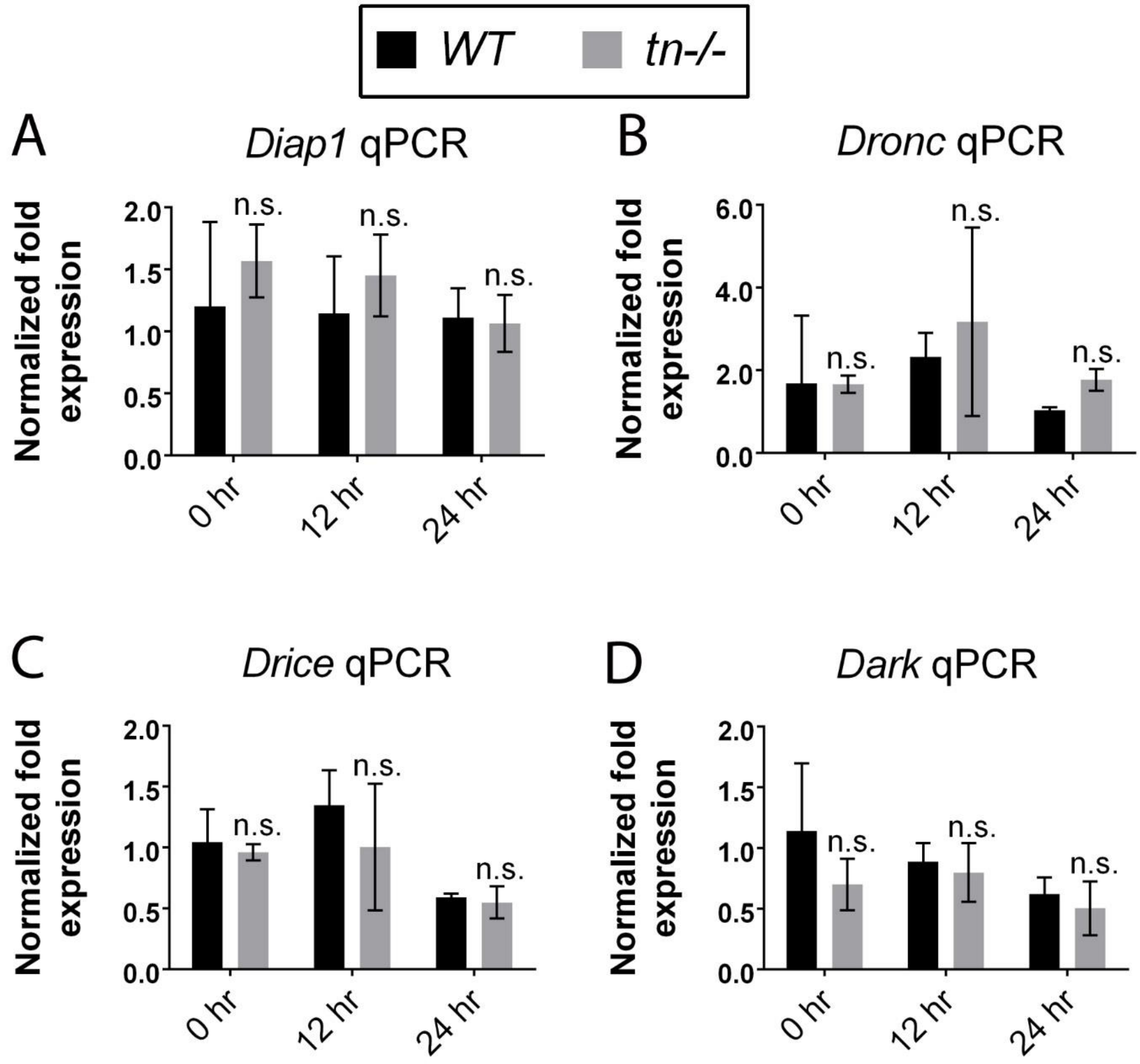

Figure S2. *tn* does not regulate the transcript levels of cell death components. (A-D) qPCR was used to assess mRNA levels from whole pupae in *WT* or *tn* mutants at the indicated time points before (0 h), during (12 h), or after (24 h) DEOM1 histolysis. There are no significant differences in the mRNA levels of *Diap1* (A), *Dronc* (B), *Dark* (C) or *Drice* (D) in *tn* -/- as compared to *WT* pupae at any stage of DEOM histolysis. Mean ± SEM, (n.s., not significant).

Vishal, et al., FIGURE S3

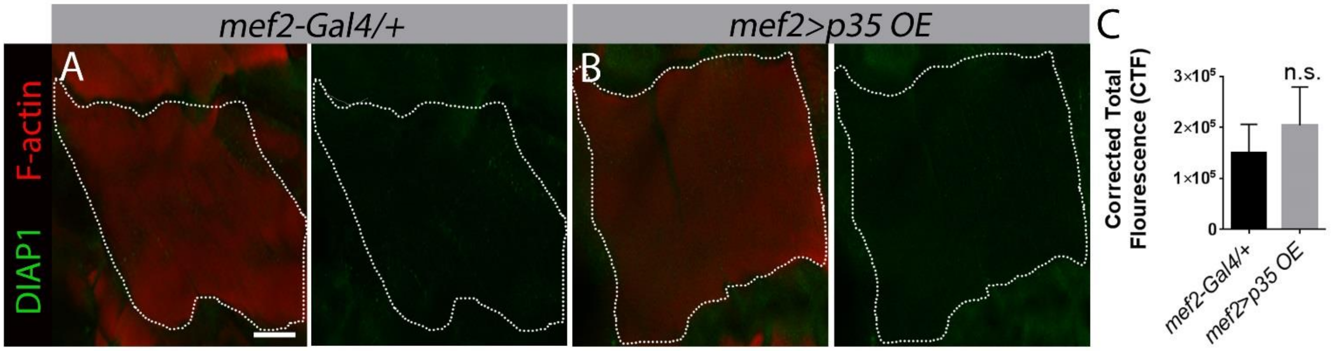

Figure S3. Overexpressing *p35* does not affect DIAP1 levels in the DEOMs. Confocal micrographs of abdominal muscles co-labeled for F-actin (red) and DIAP1 (green). (A-B) There is no significant difference in DIAP1 levels between control *mef2-Gal4/+* (A) or *mef2>p35 OE* (B) muscles at 12 h APF. (C) A bar graph showing the corrected total fluorescence of DIAP1 immunostaining in the indicated genotypes. Mean ± SEM, (n.s., not significant). Scale bar, 50μm.

Vishal, et al., FIGURE S4

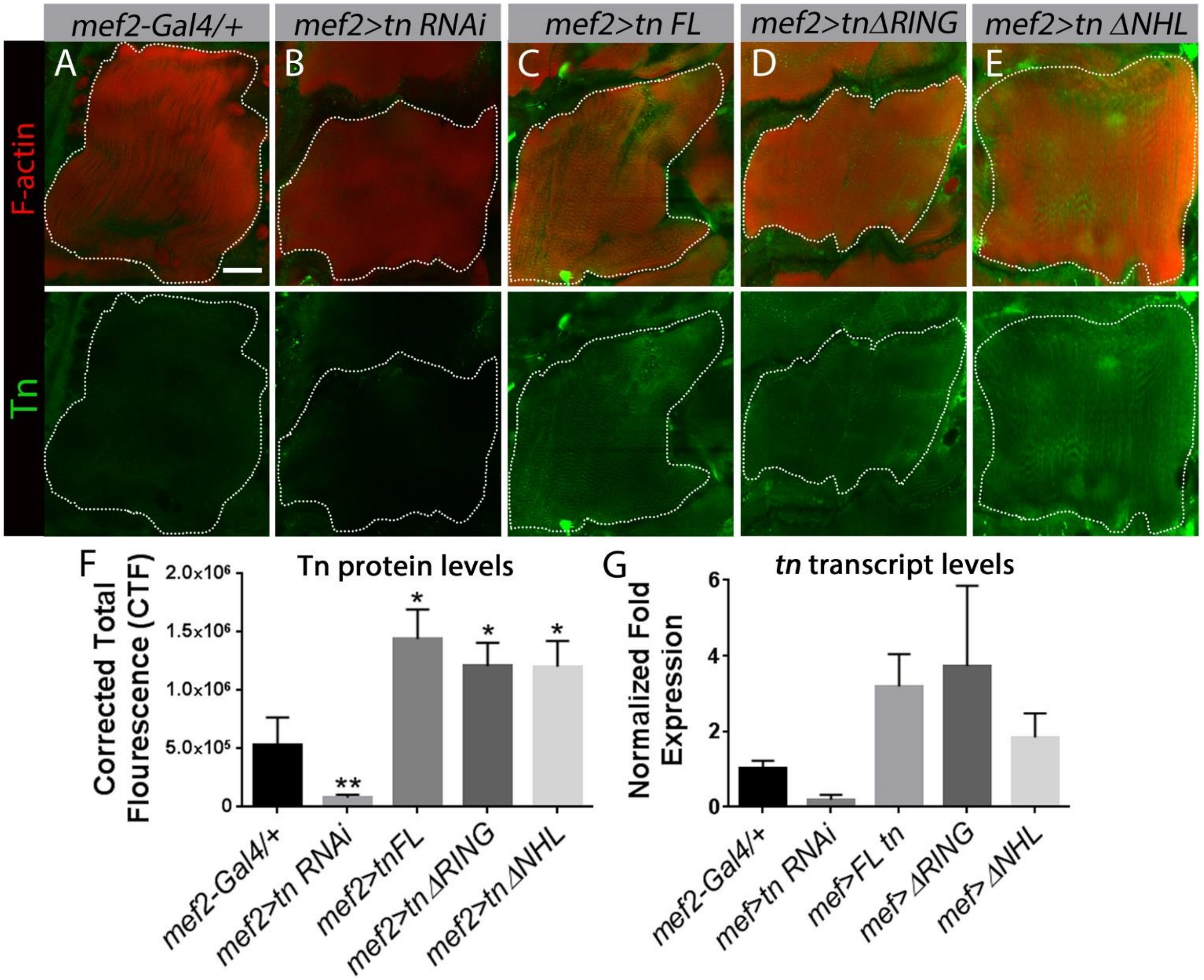

Figure S4. Expression levels of Tn protein and *tn* transcripts at 12 h APF. (A-E) Confocal micrographs of DEOMs to visualize F-actin (red) and Tn protein (green). (F) There is a significant upregulation of Tn protein levels in *mef2>tn FL*, *mef2>tnΔRING* and *mef2>tnΔNHL* DEOM muscles over *mef2-Gal4/+* control muscles or upon knockdown of *tn* using RNAi. (G) A bar graph showing upregulation of *tn* transcript levels in the indicated *UAS-tn* overexpression constructs. Mean ± SEM, (**, $p<0.01$, * $p<0.05$). Scale bar, 50μm.

Vishal, et al., FIGURE S5

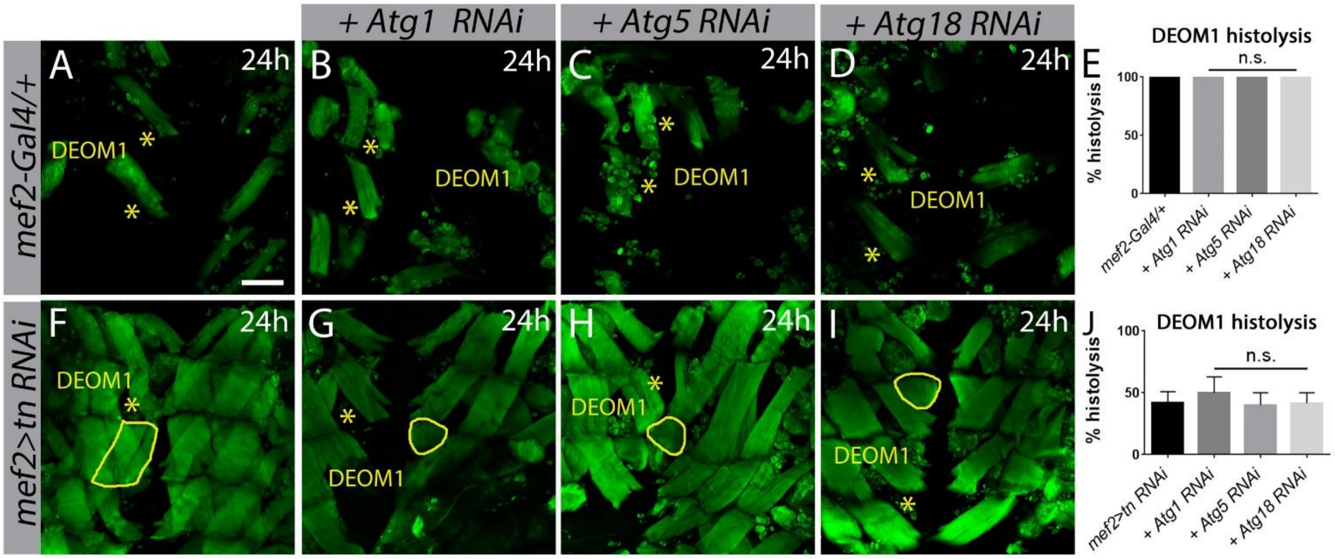

Figure S5. DEOM histolysis does not require autophagy. (A-D) There is no significant difference in DEOM1 breakdown between *mef2-Gal4/+* control (A) or *mef2>Atg1RNAi* (B), *mef2>Atg5 RNAi* (C) and *mef2>Atg 18a RNAi* (D) pupae. (E) A bar graph showing complete histolysis in all genotypes examined at 24 h APF. (F-I) DEOM degeneration is not significantly enhanced in upon a reduction in *Atg1* (G), *Atg5* (H), and *Atg18* (I) compared to *mef2;tn RNAi* alone (F). (J) Quantification of DEOM1 histolysis in (F-I). Mean ± SEM, (n.s., not significant). Scale bar, 100µm.
